# Supplementary material for: Orbital climate variability on the northeastern Tibetan Plateau across the Eocene–Oligocene transition
Source: Nat Commun. 2020 Oct 16;11:5249. doi: 10.1038/s41467-020-18824-8 (PMC7567875; doi:10.1038/s41467-020-18824-8)
Supplement: Supplementary file 1 — Supplementary Information [file 41467_2020_18824_MOESM1_ESM.pdf]

Supplementary Information

Orbital climate variability on the northeastern Tibetan Plateau across the Eocene–Oligocene transition

Ao et al.

Supplementary Note 1: Magnetostratigraphy

Characteristic remanent magnetization (ChRM) directions for 295 new samples from the Yehucheng Formation (383–570 m) together with our previously published 614 samples from the overlying Xianshuihe Formation (0–383 m, the same Duitinggou section) cluster in antipodal normal and reversed polarity groups in the equal-area stereographic projection plot (Supplementary Fig. 6). They pass a class C reversals test of McFadden and McElhinny[1] at the 95% confidence level, which supports the interpretation that they represent original ChRM directions, with only a minor influence of potential overprinting if at all. The Xianshuihe Formation (0–383 m) in the upper Duitinggou section and the coeval Xining Basin sections have consistent magnetostratigraphic correlations with the geomagnetic polarity time scale (GPTS) (Supplementary Fig. 4). Established magnetostratigraphic records from the Lanzhou[2-6] and Xining[7-12] basins are regionally consistent and supported by mammal and pollen biostratigraphy. The associated uncertainties are addressed at length in those studies, so we do not reiterate their established correlations with the GPTS. We focus on correlating our results with the GPTS. Published studies provide an important starting point for correlating the Yehucheng Formation (383–570 m) in the lower Duitinggou section to the GPTS, where the thick Xianshuihe Formation (0–383 m) in the upper Duitinggou section ranges from polarity chron C5En down to uppermost chron C12r, with ages from ~18 to 31 Ma[2,3] (Supplementary Fig. 4). Downward magnetostratigraphic continuation in the concordantly underlying Yehucheng Formation (383–570 m) in the lower Duitinggou section extends from polarity chrons C12r to C16n.1n, which is supported by a convincing correlation to the GPTS and by comparable magnetostratigraphic and lithological results from the northern Lanzhou Basin[6] and from many sections in the Xining Basin[7,8,10] (Fig. 2). In both the Lanzhou and Xining basins, cyclic gypsum beds were deposited during the late Eocene, while the Oi-1 event coincides with the end of regular alternations of gypsum layers and red mudstone/siltstone beds at the gypsum (or gypsiferous mudstone) bed marked layer B, just prior to normal polarity chron C13n (Fig. 2; Supplementary Fig. 4).

Correlation of the underlying Yehucheng Formation to polarity chrons C13r to C16n.2n would also be possible (Supplementary Fig. 4, red dashed lines); however, this alternative correlation would require a > 2.5-Myr sedimentation gap between the Xianshuihe and Yehucheng Formations to explain the missing long-duration C12r and C13n polarity chrons, which is inconsistent with the otherwise generally continuous sedimentation of the Yehucheng and Xianshuihe Formations. Furthermore, it is also inconsistent with the established upper Yehucheng Formation magnetostratigraphy from the Yongdeng section (continuous from C16n.2n to C12r) in the northern Lanzhou Basin[6] and the late Eocene magnetostratigraphy from the Xining Basin (Fig. 2). In particular, it would relate the final cyclically deposited gypsum (or gypsiferous mudstone) layer (marker layer B) to the uppermost reversed polarity chron C15r (Supplementary Fig. 4), which is inconsistent with its location in the uppermost reversed polarity chron C13r at various Xining Basin sections (Fig. 2; Supplementary Fig. 4). In addition, magnetozones R1, N2, and R2 are thicker than expected for the corresponding polarity chrons C13r, C15n, and C15r, respectively. Combining our magnetostratigraphic correlations with the GPTS and established results from the Lanzhou and Xining basins, we conclude that the Yehucheng Formation (383–570 m) is concordant with the Xianshuihe Formation in the Duitinggou section; it most likely spans continuously from polarity chrons C12r to C16n.1n. The alternative correlation option from C13r to C16n.2n is considered unlikely and would require reinterpretation of several published data sets.

Supplementary Note 2: Justification of the use of low-frequency magnetic susceptibility to assess hydroclimate variability

Thermal demagnetization curves (Supplementary Fig. 5), isothermal remanent magnetization (IRM) acquisition curve analyses (Supplementary Fig. 7), low-temperature magnetic measurements (Supplementary Fig. 8), and first-order reversal curve (FORC) diagrams (Supplementary Fig. 9) suggest the presence of hematite and magnetite/maghemite in the late Eocene–early Oligocene Lanzhou Basin terrestrial sediments. Thermal demagnetization data suggest that the characteristic remanent magnetization (ChRM) directions are isolated from 250–350 °C to 580 or 680 °C (Supplementary Fig. 5), which are consistent with the presence of both magnetite and hematite in the sediments. IRM acquisition curves and their cumulative log-Gaussian decomposition analysis (Supplementary Fig. 7) suggest two components: a strong high-coercivity component (consistent with hematite) that is associated with a low-coercivity component. The presence of magnetite is also apparent in the low-temperature remanence measurements of magnetic extracts (Supplementary Fig. 8). Low-temperature warming of a saturation IRM (SIRM) acquired at 5 K after field cooled (FC) treatments and low-temperature cycling of a room temperature SIRM all record the Verwey transition due to detrital coarse vortex state and multidomain (MD) magnetite at 120 K, which is higher than that of biogenic single domain (SD) magnetite at ~100 K[13,14]. This indicates that the coarse magnetite in the sediments is dominantly detrital rather than biogenic. The humped nature of low temperature cycling SIRM curves indicate an additional presence of maghemite[15], which likely formed via (partial) oxidation of magnetite during subaerial exposure under oxic conditions. Original samples have much lower magnetic mineral concentrations and, thus, noisier FORC diagrams than the magnetic extracts (Supplementary Fig. 9). However, they consistently suggest an absence of biogenic magnetite that produces a major central ridge signal along $B_u = 0$ with high contour density at B_c values of >10 mT[13,16]. All the magnetic extracts are characterized by a FORC peak at the origin of the diagram with large divergent outer contours (Supplementary Fig. 9), which points to the presence of detrital vortex state and MD magnetite particles[17-19]. The peak at the origin of the FORC diagram is associated with a nearly vertical distribution immediately adjacent to the B_u axis in the lower half plane (Supplementary Fig. 9), which is indicative of superparamagnetic (SP) particles[18-20]. The positive linear correlation between low-frequency magnetic susceptibility (χ_{lf}) and high-frequency magnetic susceptibility (χ_{hf}), with a rather high correlation coefficient (R = 0.83) before 33.7 Ma (Supplementary Fig. 10), also suggests a substantial SP content. Thus, it appears that the χ_{lf} record is also influenced by pedogenic SP magnetite/maghemite concentrations, in addition to coarse detrital magnetic particles.

Backscattered electron (BSE) images and elemental X-ray mapping of magnetic extracts (Supplementary Fig. 11) provide direct evidence for detrital magnetic iron oxides, without post-depositionally formed authigenic magnetic minerals (e.g., greigite). Magnetite/hematite grains are large (mostly between 3 and 20 μm) and have variable and irregular shapes (Supplementary Fig. 11); fine grains are invisible due to the limited resolution of this observation method. Nevertheless, these results provide clear documentation of detrital magnetite and hematite, consistent with magnetic measurements. Biogenic magnetite, which has well-defined nanocrystal shapes and/or chain structures[13], is absent from the BSE observations, as is also indicated by low-temperature magnetic measurements and FORC diagrams (Supplementary Figs. 8–9).

The magnetic mineralogical analyses presented above and consistent χ_{lf}, SIRM, and hard IRM (HIRM) variations with positive linear correlations (Supplementary Figs. 5 and 7–12) suggest that χ_{lf} of playa-palaeolake deposits in the Lanzhou Basin reflects primarily magnetic mineral concentration changes. As observed in studies of the Xining Basin sedimentary, clay mineral, and pollen records[8,10,21-23], low χ_{lf} units with low magnetic mineral concentrations are related to high regional precipitation and a relatively deep/large lake, and *vice versa*. This relationship between χ_{lf} and climate/lake conditions also holds in the coeval Lanzhou Basin that had similar late Eocene–early

Oligocene hydroclimate and hydrological conditions because both Lanzhou and Xining basins were part of the larger Longzhong Basin[5,12]. The observed relationship between χ_{lf} and hydroclimate is common in fluvial-lacustrine sediments[17,24-26]. In the Lanzhou Basin, the greyish white gypsum and siltstone beds that formed under perennial anoxic playa lake to alluvial subaqueous conditions, generally have lower χ_{lf} values than the red mudstone beds, which formed under distal alluvial fan conditions. Generally, wet periods promoted perennial playa lake to alluvial anoxic subaqueous conditions, which could have facilitated partial post-depositional detrital magnetic mineral dissolution[17,24] and authigenic formation of non-magnetic gypsum, thus diluting the magnetic signal (Supplementary Fig. 13). Moreover, in a wetter climate the playa lake was larger with higher lake levels; clastic materials were probably transported rapidly to the lake from its catchment regions. Subaerial exposure was likely limited as was pedogenic magnetic mineral formation, while anoxic subaqueous depositional conditions prevailed in the shallow playa lake[8,22]. Weak subaerial pedogenic magnetic mineral formation together with potential magnetic mineral dissolution during subsequent anoxic subaqueous depositional conditions in the playa lake as implied by their greyish white colour may have caused their low χ_{lf} values. The combined dissolution, dilution, and near-absence of pedogenic effects, thus, provide a plausible interpretation for the low χ_{lf} values in the greyish white gypsum and siltstone beds. The occurrences of several fine-grained sand beds with large mean grain sizes (Fig. 2) were probably due to catastrophic flood events, which drove extremely rapid clastic transportation to the playa lake, with minimal subaerial pedogenesis that is consistent with their greyish white colour. In contrast, dry periods induced playa lake retreat, which increased the time available for pedogenic magnetic mineral formation under mudflat conditions[22]. Moreover, shallower lake levels also tend to promote oxic distal alluvial fan conditions with frequent subaerial exposure, which may have facilitated detrital magnetic mineral preservation during deposition and even sustained pedogenic magnetic mineral formation during subaerial exposure (Supplementary Fig. 13). The χ_{lf} and χ_{fd} records have a higher correlation coefficient and vary consistently in larger amplitudes before 33.7 Ma (Supplementary Figs. 10 and 12). This suggests that the subaerial pedogenesis had a significant influence on χ_{lf} variability, particularly before Oi-1 when contrasting conditions alternated periodically between perennial lakes and alluvial fans. In addition, gypsum beds disappeared with decreased dilution of non-magnetic materials. These combined processes likely resulted in relatively higher χ_{lf} values in the red mudstone beds.

The Lanzhou Basin fluvial-lacustrine sediments have low χ_{lf} values (~10^{-7}–10^{-8} m^3/kg). They are lower than early Pleistocene loess χ_{lf} values (which are in the order of 10^{-7} m^3/kg) in the Lanzhou Basin[27], and significantly lower than Pleistocene loess χ_{lf} values (~10^{-6}–10^{-7} m^3/kg) on the eastern Chinese Loess Plateau[28]. This points to low late Eocene–early Oligocene regional precipitation when Asian monsoon moisture transportation was weak and the Tibetan Plateau probably was already sufficiently elevated to produce a rain shadow effect[29-31]. This indicates that subtle changes in pedogenic, dissolution, dilution, and preservation effects would have driven notable χ_{lf} changes in the Lanzhou Basin fluvial-lacustrine sediments. Pedogenesis during subaerial exposure caused SP magnetic mineral formation, the concentration of which can be measured by χ_{fd}. Despite low values, the χ_{lf} and χ_{fd} are positively correlated and vary consistently (Supplementary Figs. 10 and 12). This supports our inference that the pedogenesis influenced the magnetic mineral concentration. In addition, FORC diagrams, low-temperature magnetic measurements, and mineralogical and morphological analyses suggest that biogenic magnetite and authigenic iron sulfides are absent in the Lanzhou playa-palaeolake sediments. Based on the above observations and reasoning, regular (orbital) χ_{lf} alternations between high and low values throughout the late Eocene to early Oligocene was likely caused by a combination of subtle climate-induced changes in pedogenic, dissolution, dilution, and

preservation effects, which can be linked to orbital hydroclimate and lake condition variability. Cyclical geological records from the Lanzhou palaeolake provide sensitive high-resolution archives of late Eocene–early Oligocene NE Tibetan Plateau climate changes, which are comparable to those in the coeval neighouring Xining Basin playa-palaeolake records (e.g., Figs. 2 and 3).

Supplementary Note 3: Age model uncertainty and orbital variability justification

Compared to Quaternary astronomical time scales[32-34], the late Eocene–early Oligocene Duitinggou astronomical time scale has large uncertainties. Among the 42 correlation points, 20 have a ≥80-kyr negative uncertainty, and 23 have a ≥80-kyr positive uncertainty (red vertical lines in Supplementary Fig. 16f; Supplementary Table 1). These uncertainties are generally associated with variable eccentricity correlations, which delay or predate our selected correlation usually by about a 100-kyr short eccentricity cycle and occasionally by two 100-kyr short eccentricity cycles. Uncertainties below 80 kyr are generally associated with moderately and slightly different eccentricity correlations in our selected option, with potential obvious differences over obliquity and precession time scales in some intervals. The smallest negative and positive uncertainties are -10 and +20 kyr, respectively. Such large age uncertainties are common in old palaeoclimate records (e.g., late Eocene–early Oligocene records), which are > 30 Myr before present and are more difficult to be dated precisely than records from the last 5 Myr. Likewise, moderate uncertainties are also present in ages for the late Eocene–early Oligocene GPTS (Supplementary Table 2) and marine benthic $\delta^{18}O$ records. The age of the C13n–C12r reversal boundary within the Oi-1 event is ~160-kyr younger than its 2012 GPTS age[35] and ~80-kyr younger than its 2020 GPTS age[36] (Supplementary Table 2). Decreasing these uncertainties requires development of both a more precise GPTS and further continuous high-resolution marine/terrestrial records. However, the uncertainties do not influence the observed major orbital hydroclimate transition across Oi-1 because they are also apparent in the original depth domain (Supplementary Fig. 14) and in the untuned magnetochronology (Supplementary Fig. 15). The age uncertainty of the observed climate transition across Oi-1 in the terrestrial χ_{lf} and marine benthic $\delta^{18}O$ records is much less than the typical uncertainties determined here because it coincides with the C13r–C13n reversal boundary.

To avoid over-tuning, we did not further tune our eccentricity-based astronomical time scale to obliquity and precession cycles, particularly because these shorter cycles are absent in the Lanzhou Basin χ_{lf} record in the original depth domain and in the untuned magnetochronology before 33.7 Ma (Supplementary Figs. 14–15). The absence of obliquity and precession tuning resulted in age uncertainties for these shorter cycles, which likely weakened their expression and resulted in non-orbital bands around the obliquity and/or precession bands. However, we note that obliquity and precession are still clear after 33.7 Ma in the eccentricity-based astronomical time scale (Fig. 4), in the original depth domain (Supplementary Fig. 14), and in the untuned magnetochronology (Supplementary Fig. 15). This indicates that the related obliquity and precession cycles are robust enough not to be removed by accompanying age uncertainties. Age uncertainties and subtle lithological variability may have caused spectral noise (i.e., non-orbital signals) in the orbital climate expression, which could attenuate the intensity and continuity of the orbital signature in spectral evolution diagrams (Fig. 4; Supplementary Figs. 14–15). However, the uncertainties did not change the observed major orbital pattern, which is observed consistently in the χ_{lf} record using the eccentricity-based astronomical time scale, the untuned magnetochronology, and the original depth domain (Fig. 4; Supplementary Figs. 14–15). Our minimum eccentricity tuning strategy decreased age uncertainties and intensified the untuned obliquity and precession bands relative to the magnetochronology, so their expression is apparently real.

The Duitinggou lacustrine section is generally continuous (complete) from the late Eocene to early Oligocene and does not contain appreciable hiatuses or redeposited intervals that may potentially obscure (or "shred") sedimentary environmental signals[37]. Compared to the various late Eocene to early Oligocene GPTS age models, our age model has uncertainties <400 kyr (Supplementary Table 2). Our first-order tuning of the 405-kyr eccentricity band, which is the longest and most stable late Eocene–early Oligocene climate rhythm[38-45], and least likely to be missed, allows the tuned age model to be accurate over these cycles within magnetochronological constraints. Distinct spectral bands with a period between ~1.5 and 1.9 m that corresponds to the obliquity, and between ~0.7 and 1 m that corresponds to precession (~19 kyr, ~23 kyr), also appear above ~500 m (after ~33.7 Ma) in the depth domain (Supplementary Fig. 14). Appearance of obliquity and precession cycles after ~33.7 Ma is also apparent from the untuned magnetochronology (Supplementary Fig. 15). These untuned results support the robustness of an orbital response shift at ~33.7 Ma, and exclude artifacts due to the tuning procedure. Moreover, since we used eccentricity rather than obliquity and precession to tune, features of obliquity and precession variability are unlikely to result from the tuning procedure. In addition, use of 3-point and 5-point running χ_{lf} means, which reduced short-term noise in the original χ_{lf} record, does not affect the appearance of obliquity and precession after Oi-1 (Supplementary Fig. 17b–c). This testifies to the robustness of the observed orbital response shift across Oi-1. Due to larger smoothing effects, the precession signal is smoothed away by the 10-point running χ_{lf} mean, which is associated with a weak obliquity signal after Oi-1 (Supplementary Fig. 17d).

The absence of conglomerate and coarse sand layers in the late Eocene–early Oligocene Duitinggou lacustrine section supports the absence of significant sedimentary erosion and/or hiatuses. Potential small hiatuses could attenuate, distort, or (partially) remove precession and obliquity cycle expression over short intervals. This may apply particularly to the age range in our record before ~33.7 Ma when sedimentation rates were lower than after 33.7 Ma. However, such disruptions are not evident through the late Eocene–early Oligocene Duitinggou section as suggested by detailed sedimentological observations, including absence of root and burrow traces, and rare small-scale (centimetre) crossbedding. In particular, potential short-duration hiatuses do not interfere with generally clear orbital variations in the Lanzhou Basin χ_{lf} record, including the absence of strong obliquity and precession cycles throughout a long interval between 35.5 and 33.7 Ma and a shift from dominantly eccentricity pacing to combined eccentricity, obliquity, and precession pacing immediately after 33.7 Ma, although occasional unidentified small hiatuses or sedimentation rate decreases might cause one or several precession cycles to be partially missed in a few intervals. Moreover, despite lower sedimentation rates before 33.7 Ma, the sampling resolution of the χ_{lf} record still varies between ~2 and 5 kyr, which is suitable to resolve obliquity and precession cycles if they are evident. Absence of such expression before 33.7 Ma contrasts markedly with the younger portion of the χ_{lf} record, after 33.7 Ma, where precession cycles are prominently present for an only slightly higher sampling resolution (1–3.5 kyr).

Environmental smoothing related to post-depositional diagenesis and biological disturbance[46-48] in the Lanzhou palaeolake may have been stronger before 33.7 Ma, when the lake was larger and deeper and sedimentation rates were lower than after this time, so a potential smoothing influence before 33.7 Ma should be assessed. Based on the observations and comparisons below, we document that smoothing is unlikely to have erased obliquity and precession cycles throughout a long (~2 Myr) interval between 35.5 and 33.7 Ma. First, strong bioturbational mixing is generally associated with profound modification of sedimentary structure, texture, and composition[49]. An absence of root and burrow marks, and preservation of centrimetric laminar mudstone and gypsum beds before 33.7 Ma (Supplementary Fig. 3d–g) indicates that environmental smoothing was minimal. Moreover,

bioturbation is largely restricted to a narrow depth of surficial sediments, which varies from <3 cm in varved and laminated lake sediments to ~12 cm in homogenous massive lake sediments in wet regions[47,48]. A worldwide mean surface mixing depth of 5.8 ± 5.7 cm has been estimated in marine sediments[50]. The Lanzhou playa-palaeolake sediments in arid western China, where organic contents were low and bioturbation was weak, are likely to have only small bioturbational mixing depths, likely <3 cm. This depth is far smaller than the expected ~70–100 cm thickness for a precession cycle and ~150–190 cm for an obliquity cycle (Supplementary Fig. 14). Thus, even if environmental smoothing occurred, it would influence only a small part of a precession cycle, so it cannot be the cause of the absence of apparent precessional expression throughout a ~2-Myr interval between 35.5 and 33.7 Ma. The obliquity cycle is about twice as long as the precession cycle, so the effect of environmental smoothing on obliquity will be even smaller. Thus, the orbital shift observed in the Lanzhou Basin χ_{lf} record cannot be a result of sedimentation rate and smoothing variations. We conclude that it reflects a terrestrial climate reorganization.

Our spectral analysis of the Xining Basin χ_{lf} record also suggests an orbital shift at ~33.7 Ma from eccentricity dominated cycles to oscillations paced by a combination of eccentricity and obliquity (Supplementary Fig. 19). Importantly, in contrast to increased sedimentation rates across Oi-1 in the Lanzhou Basin, sedimentation rates decreased across Oi-1 in the Xining Basin[10]. Sedimentation rates averaged ~4 cm/kyr between 35.5 and 33.7 Ma and decreased to ~3 cm/kyr after 33.7 Ma, based on the established magnetochronology[10] using the 2012 GPTS[35] (Fig. 2). If an environmental smoothing effect can explain the observed orbital shift in the Xining Basin, then the obliquity expression would be attenuated after 33.7 Ma, which is not observed (Supplementary Fig. 19). Rather, the obliquity expression is absent in the Xining Basin χ_{lf} record before 33.7 Ma when sedimentation rates were higher and climate was warmer and wetter, but it appears after 33.7 Ma when sedimentation rates were lower and climate was substantially colder and drier. Thus, the Xining data provide additional support for our interpretation of the observed orbital response shift in both the Lanzhou and Xining basins in terms of an Asian climate reorganization across Oi-1. This shift is not a result of sedimentation rate variations and/or environmental smoothing effects.

In addition to the Xining Basin χ_{lf} and lithologic records, the Maoming Basin lithologic data[51], the ODP Site 1218 benthic $\delta^{18}O$ record[40,52], and high-resolution Si, Ca, and Fe records[45] from ODP Sites 1218, U1333, and U1334 also suggest an absence of evident precession and obliquity cycles between 35 and 33.7 Ma, but reveal dominant eccentricity cycles instead. Based on the evidence above, we conclude that the absence of evident precession and obliquity expression before 33.7 Ma in the Lanzhou Basin χ_{lf} record is unlikely to have been caused by sedimentation rate and environmental smoothing variations or by unidentified small hiatuses. As summarized in a flowchart of our tuning procedure in Supplementary Fig. 20, the orbital χ_{lf} shift at ~33.7 Ma is observed consistently in the depth domain (Supplementary Fig. 14), the magnetochronology (Supplementary Fig. 15), and the astronomical chronology (Fig. 4). In all three records, the orbital expression is similar, which excludes the possibility that the orbital shift at 33.7 Ma is a tuning-related artifact. Orbital tuning has refined the age model, and enhanced the recorded orbital expression, which becomes more distinct (which is the point of tuning), but it has not changed the general orbital features, particularly the orbital shift at 33.7 Ma. Importantly, this orbital shift is also evident in the Xining χ_{lf} record[10], the Maoming lithology[51], and the ODP Site 1218 benthic $\delta^{18}O$ record[40,52], which supports our interpretation of the Lanzhou Basin χ_{lf} record as an orbitally forced climate proxy record, and its robustness for understanding orbital NE Tibetan Plateau climate changes across the global Oi-1 event (Supplementary Fig. 20).

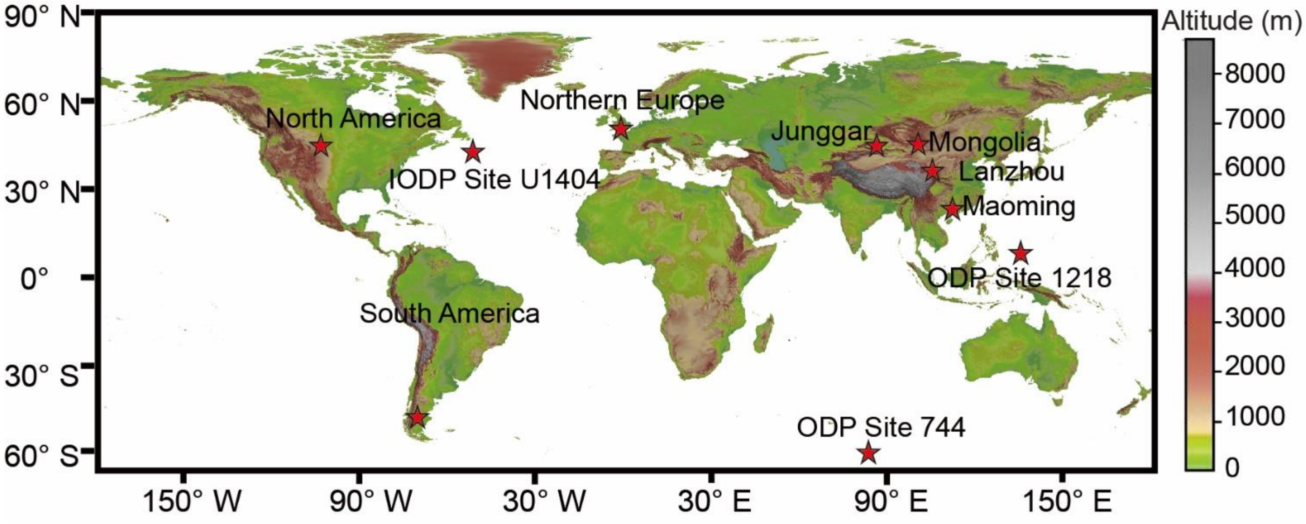

Supplementary Figure 1. Site locations of Eocene–Oligocene transition records. Locations of the Eocene–Oligocene transition (EOT) sites mentioned in the text are shown in the global topographic map with red stars. Terrestrial EOT records are rare and have been reported mainly from central North America, southernmost South America, Northern Europe, and Asia.

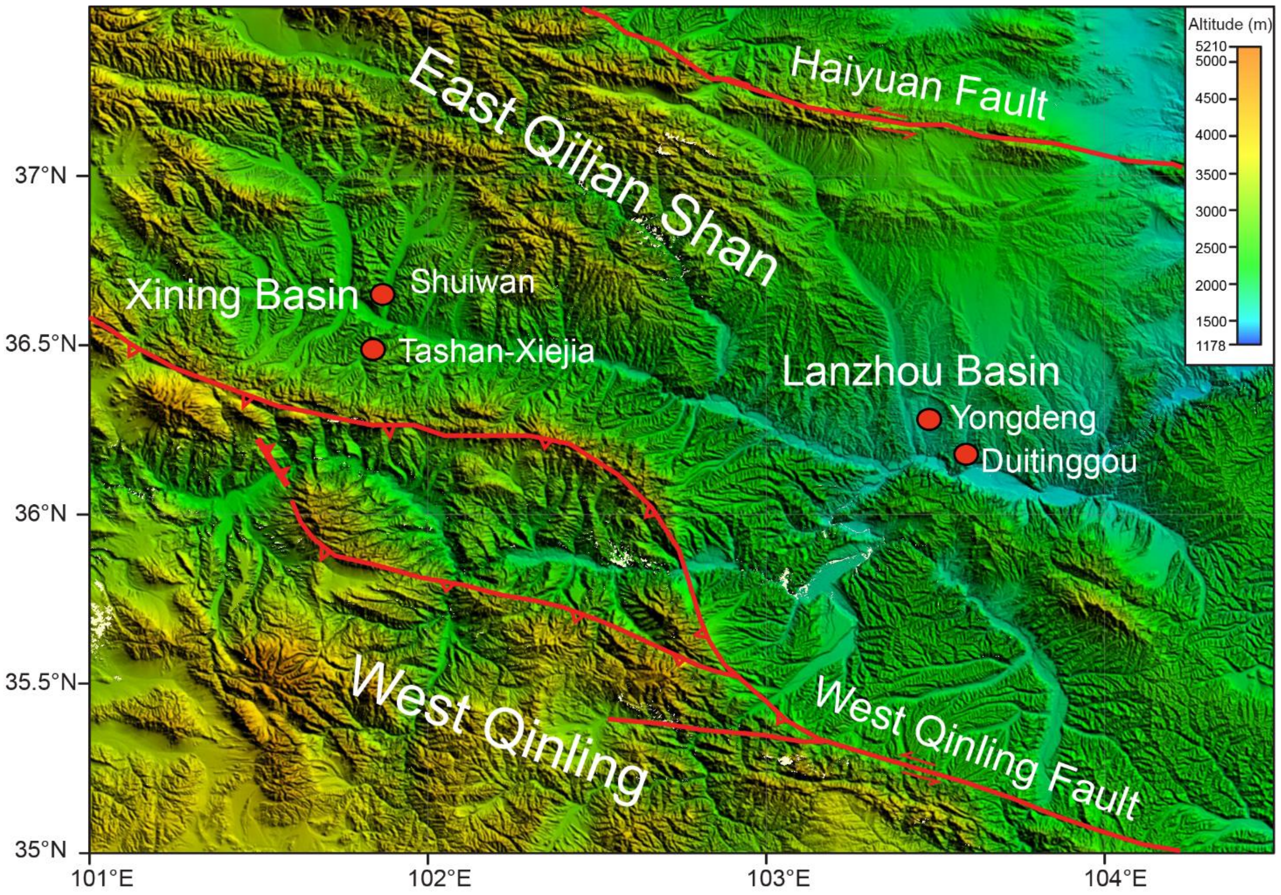

Supplementary Figure 2. Lanzhou Basin topography. The topographic map contains the Lanzhou and Xining basins, the surrounding mountains, major faults, and locations of the sections (red solid circles) mentioned in the text.

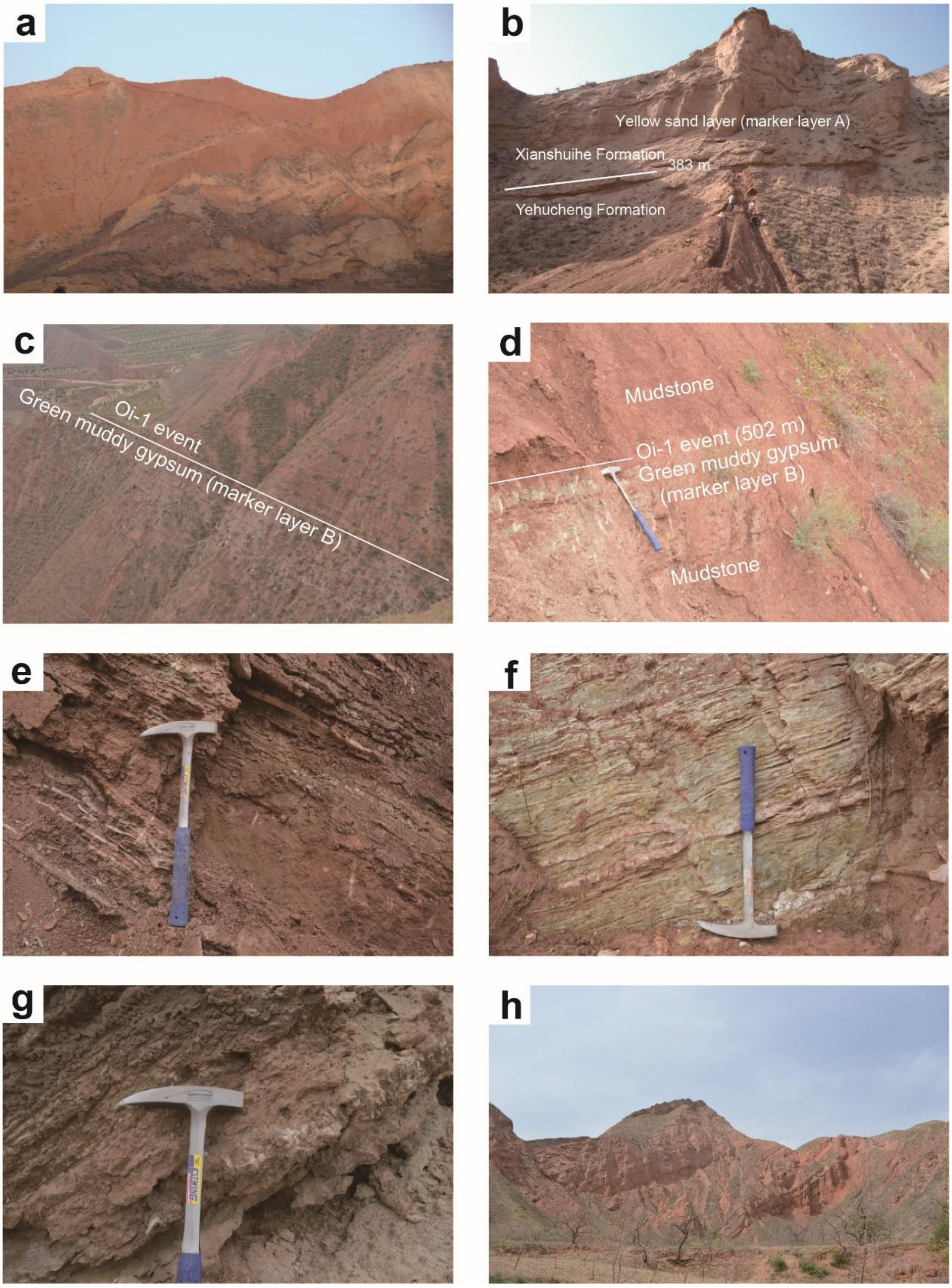

Supplementary Figure 3. Field photographs of lithologies in the Duitinggou section. (**a**) The middle Xianshuihe Formation, which consists mainly of red mudstones intercalated with yellow sandstone or conglomerate packages, (**b**) the yellow sand bed with the Nanpoping Fauna at the bottom of the Xianshuihe Formation, which is a prominent marker layer (layer A) across the Lanzhou Basin and marks the boundary between the Yehucheng and Xianshuihe Formations, (**c**) distant and (**d**) close-up views of the Oi-1 event in the sedimentary succession, which coincides with the end of regular alternations of gypsum layers and red mudstone/siltstone beds at the marked gypsum layer B, (**e**) alternations of greyish-green gypsiferous and red mudstone beds, (**f**) densely-packed green laminated gypsum bed, (**g**) beds of euhedral gypsum crystals that mimic rosette-like structures, and (**h**) the upper Xiliugou Formation with uniform red sandstones.

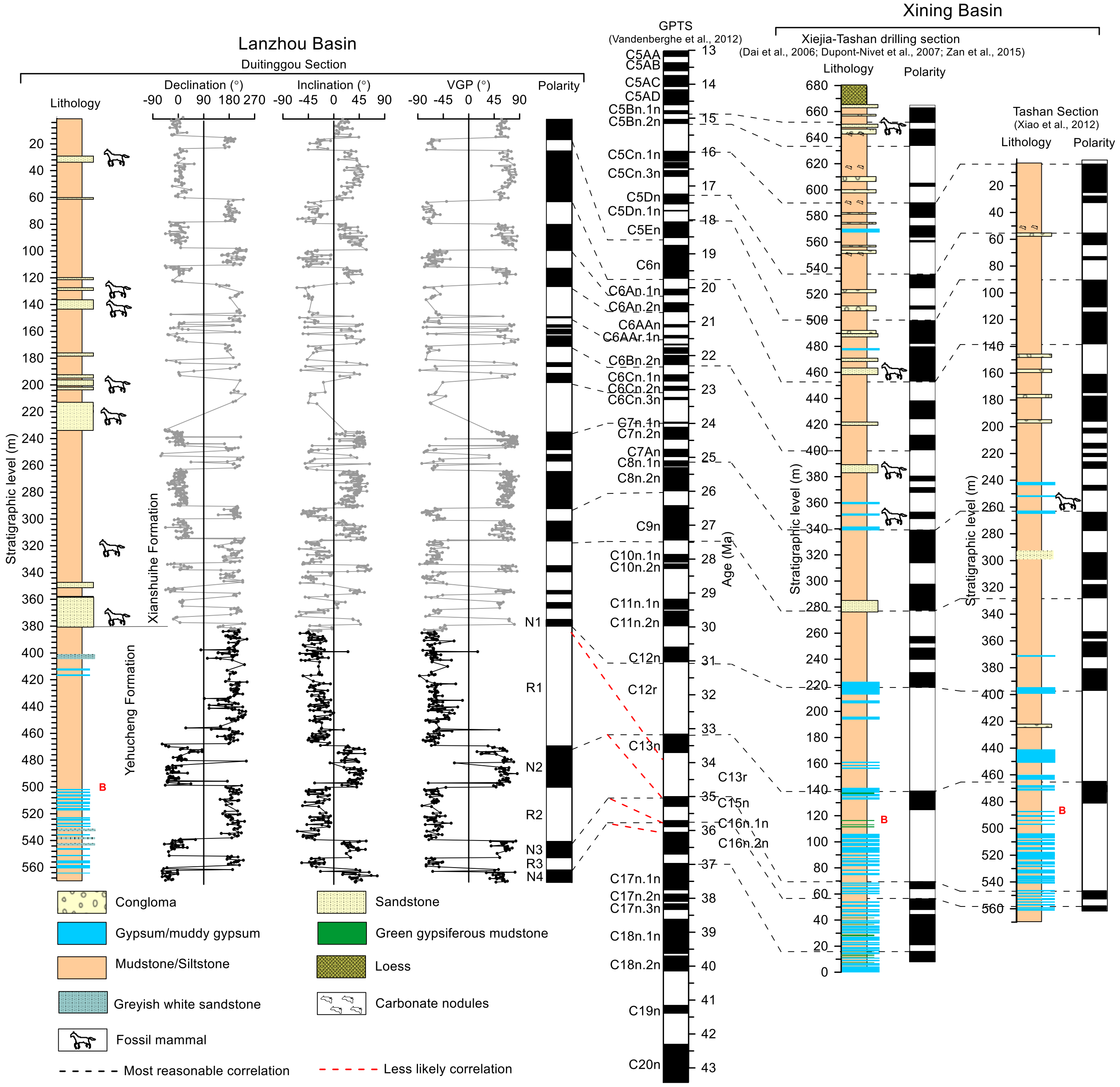

Supplementary Figure 4. Regional stratigraphic correlation. Lithostratigraphy and magnetostratigraphy of the Duitinggou section, Lanzhou Basin is comparable to that of the Xiejia[7-9] and Tashan[11] sections in the Xining Basin. Grey dots and polarity above 383 m (Xianshuihe Formation) for the Duitinggou section are from Zhang et al.[2,3]

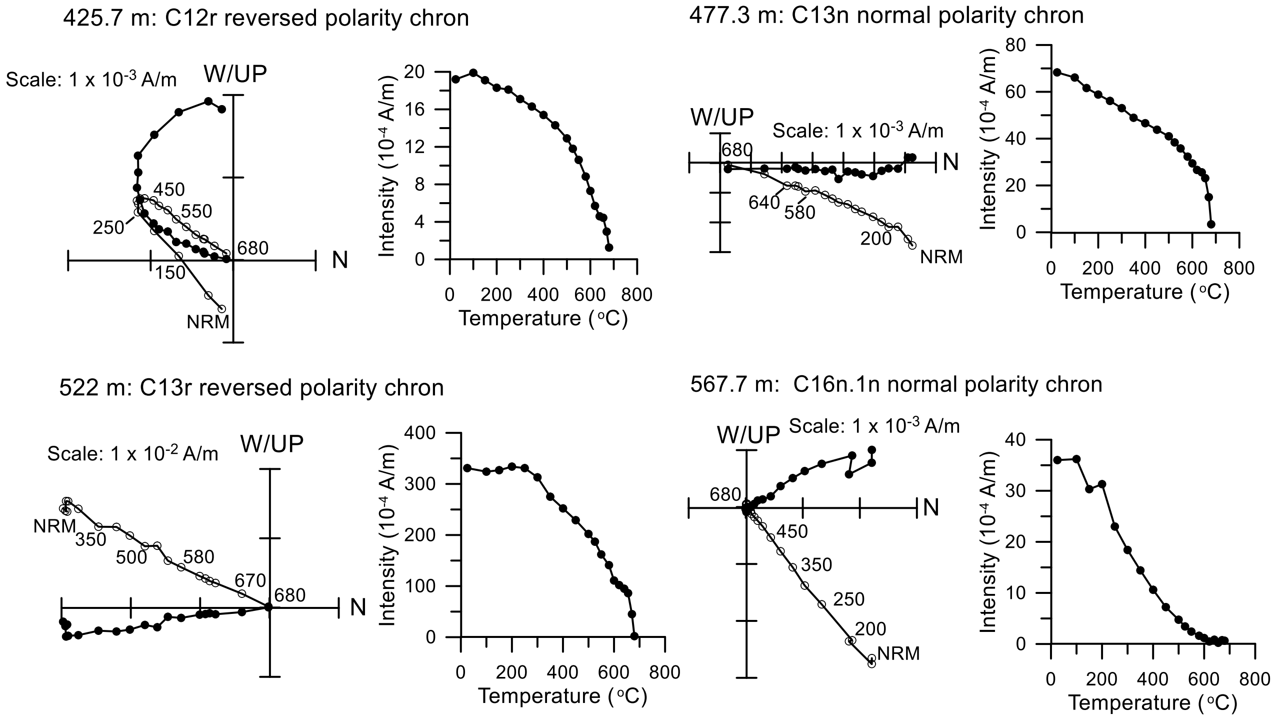

Supplementary Figure 5. Thermal demagnetization data. The plot contains vector end-point projections and intensity changes of natural remanent magnetization (NRM) demagnetization data for four selected samples from the Duitinggou section. Solid (open) circles represent projections onto the horizontal (vertical) plane[53], with thermal treatment steps indicated in °C next to open circles.

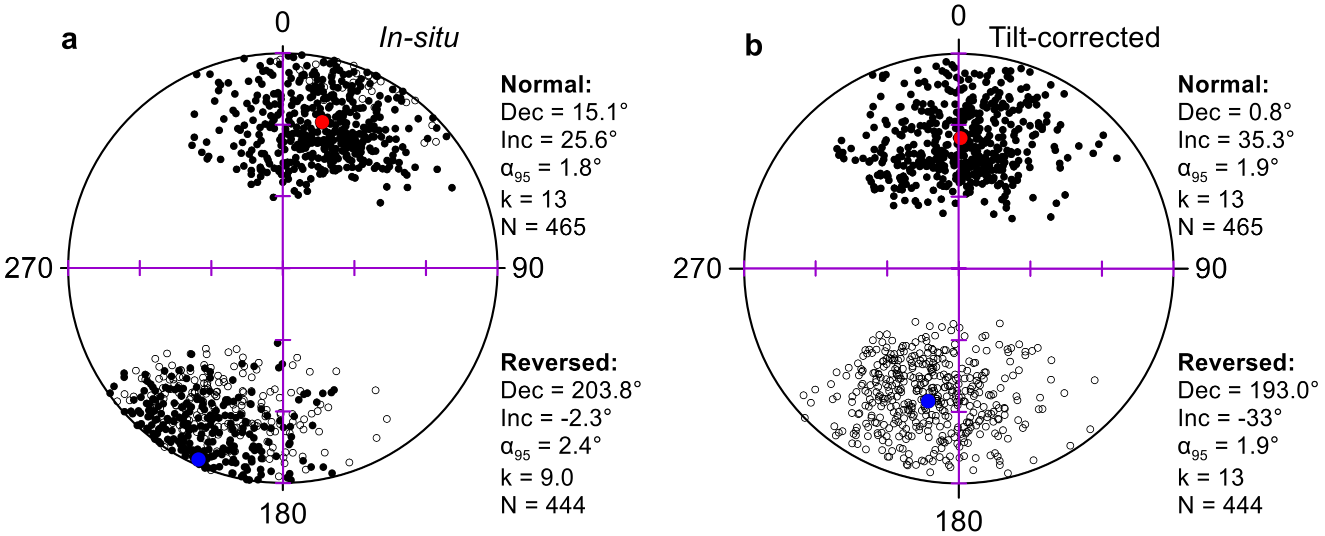

Supplementary Figure 6. Palaeomagnetic data of the Duitinggou section. (**a**, **b**) Equal-area stereographic projection with 909 characteristic remanent magnetization (ChRM) directions (614 from the overlying Xianshuihe Formation[2,3] and 295 from the Yehucheng Formation) of the Duitinggou section before (*in-situ*) and after tilt correction (tilt-corrected). Solid (open) circles represent downward (upward) inclinations. Red and blue circles in the upper and lower hemispheres represent means of normal and reversed polarity ChRM directions, respectively. ChRM directions cluster in antipodal normal and reversed polarity orientations and pass a class C reversals test of McFadden and McElhinny[1] at the 95% confidence level.

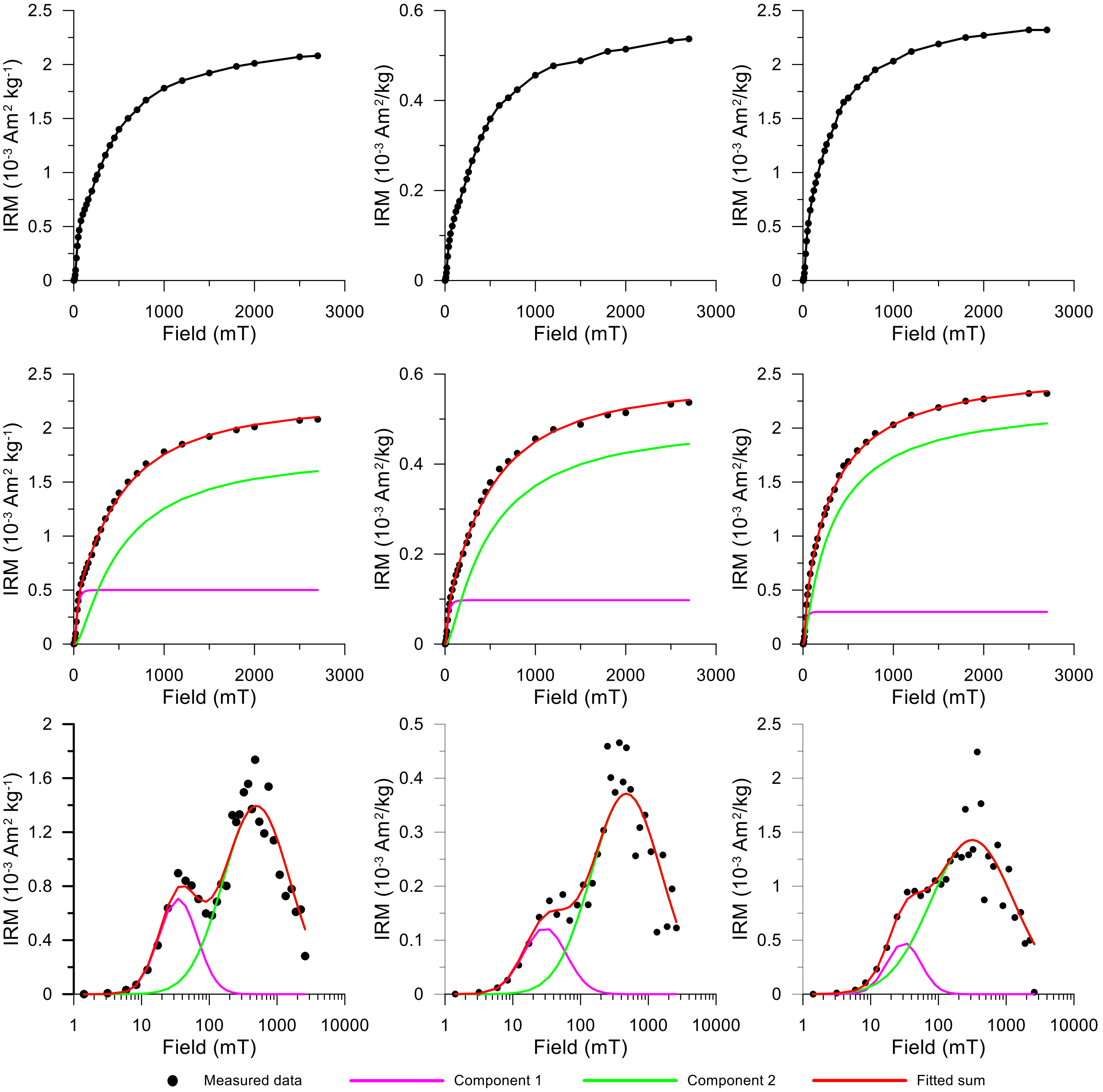

Supplementary Figure 7. Quantification of magnetic coercivity components. Magnetic coercivity components were quantified by the analysis of isothermal remanent magnetization (IRM) acquisition curves for selected samples from the Duitinggou section. The IRM gradually increases with field strength; it remains unsaturated in fields up to 2.7 T, consistent with the presence of hematite. Cumulative log-Gaussian decomposition analysis of IRM acquisition curves[54] further suggest two components: a low-coercivity component and a high-coercivity component. The IRM acquisition curve analyses reveal a strong dominance of high-coercivity hematite over low-coercivity magnetite in the sediments.

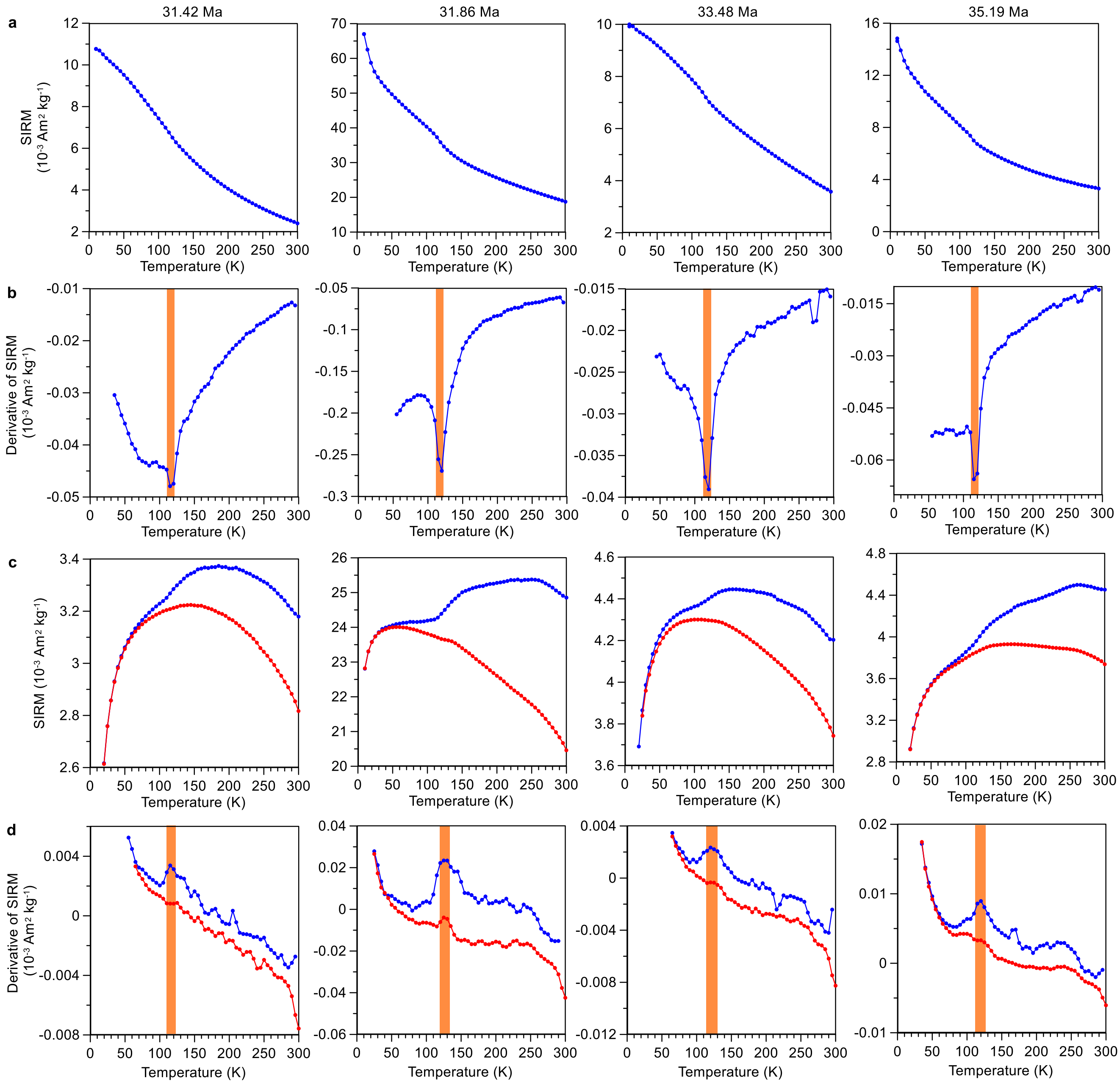

Supplementary Figure 8. Low-temperature magnetic data. Low-temperature magnetic properties were measured for magnetic mineral extracts from selected samples from the Duitinggou section. (**a**, **b**) Low-temperature warming curves of a field-cooled (5 T) SIRM warming and their derivative curves. (**c**, **d**) Low-temperature cycling of a room temperature SIRM and their derivative curves. Orange bars indicate the temperature interval of the Verwey transition in magnetite. SIRM: saturation isothermal remanent magnetization.

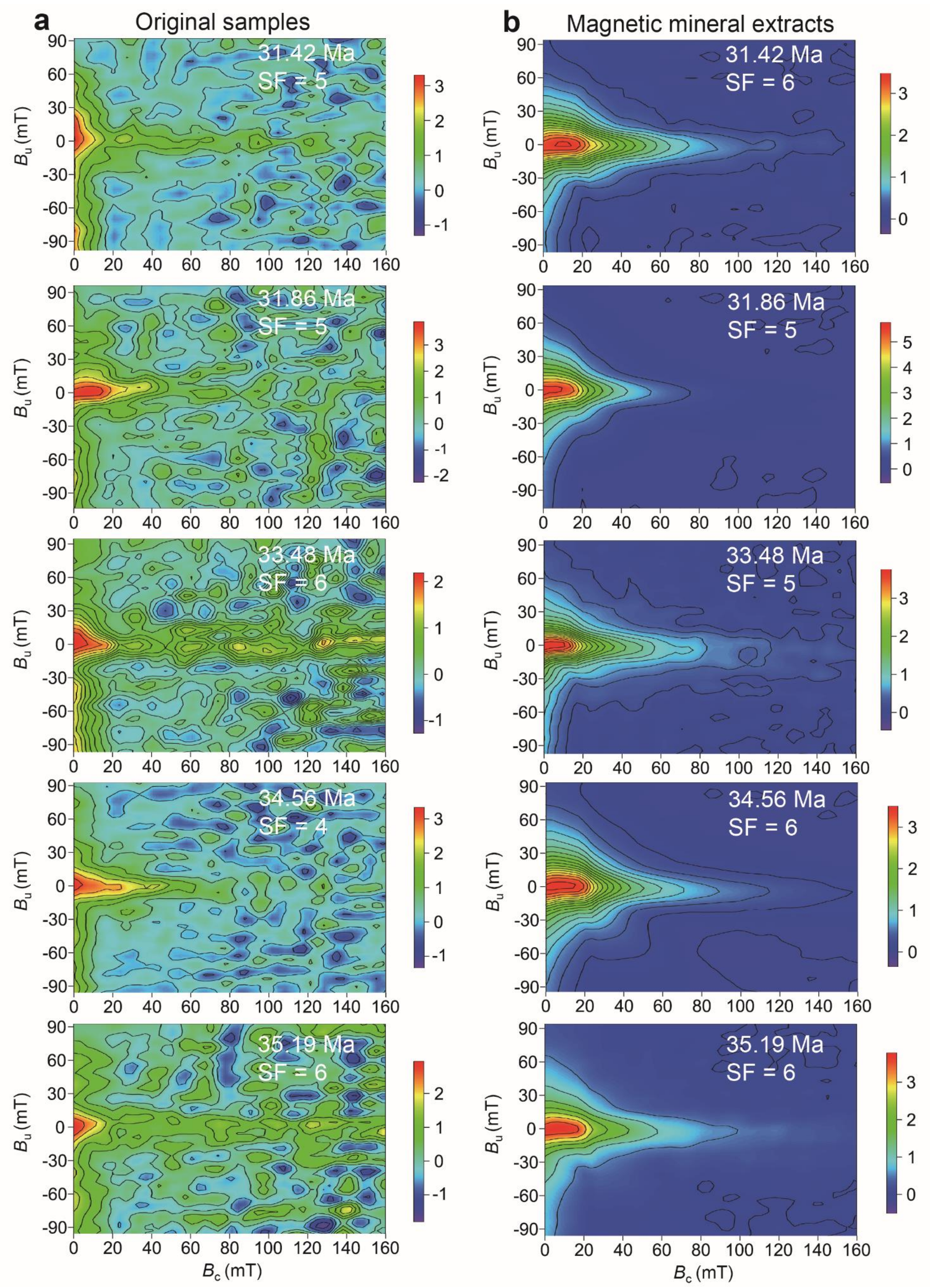

Supplementary Figure 9. First-order reversal curve diagrams. (**a**, **b**) First-order reversal curve (FORC) diagrams for selected samples from the Duitinggou section and their magnetic mineral extracts. SF: smoothing factor.

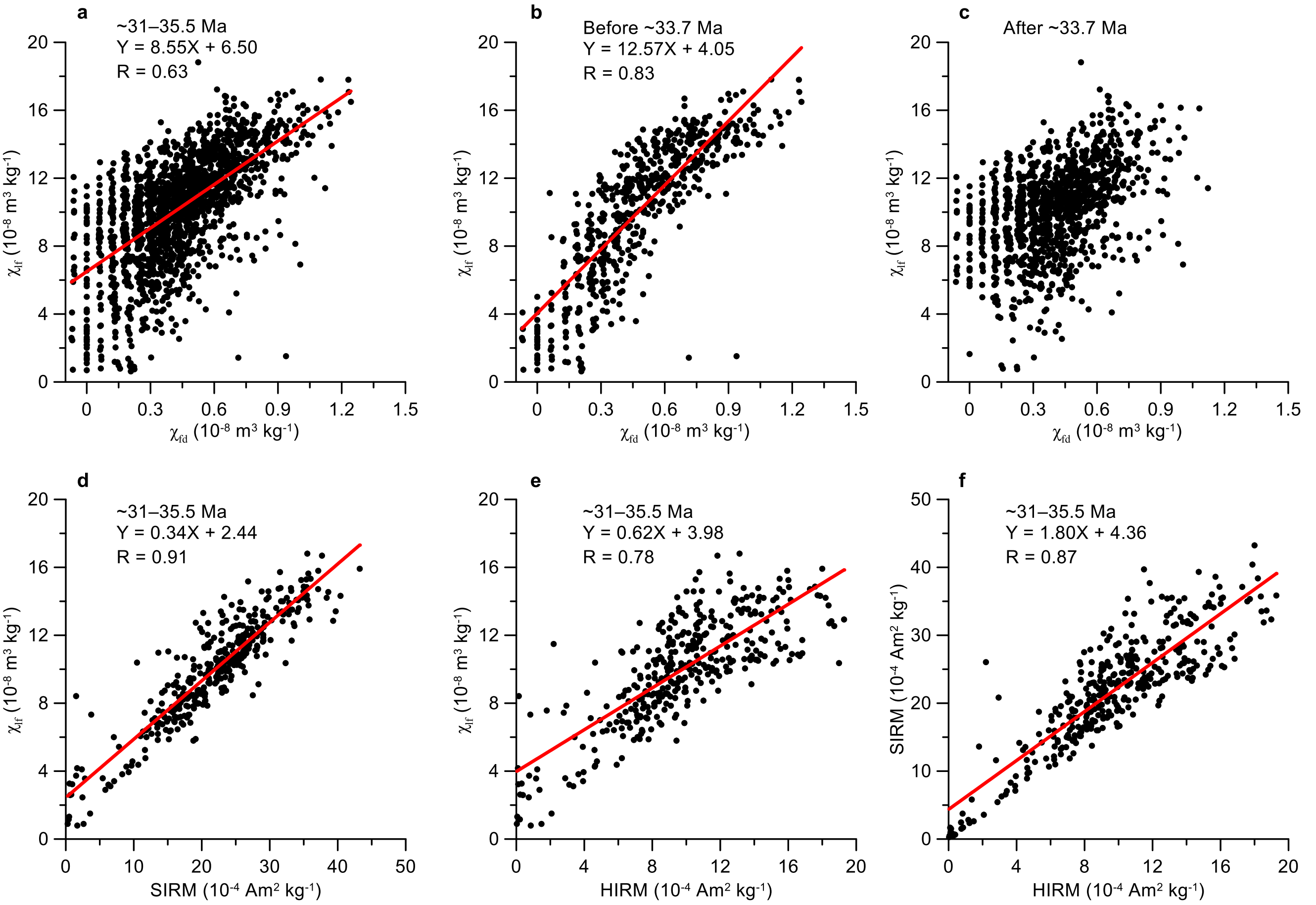

Supplementary Figure 10. Relationships among magnetic parameters for the Duitinggou section. Relationship of (**a**–**c**) χ_{lf} versus χ_{fd}, (**d**) χ_{lf} versus SIRM, (**e**) χ_{lf} versus HIRM, and (**f**) SIRM versus HIRM. χ_{lf}: low-frequency magnetic susceptibility; χ_{fd}: frequency-dependent magnetic susceptibility, SIRM: saturation isothermal remanent magnetization; HIRM: hard isothermal remanent magnetization.

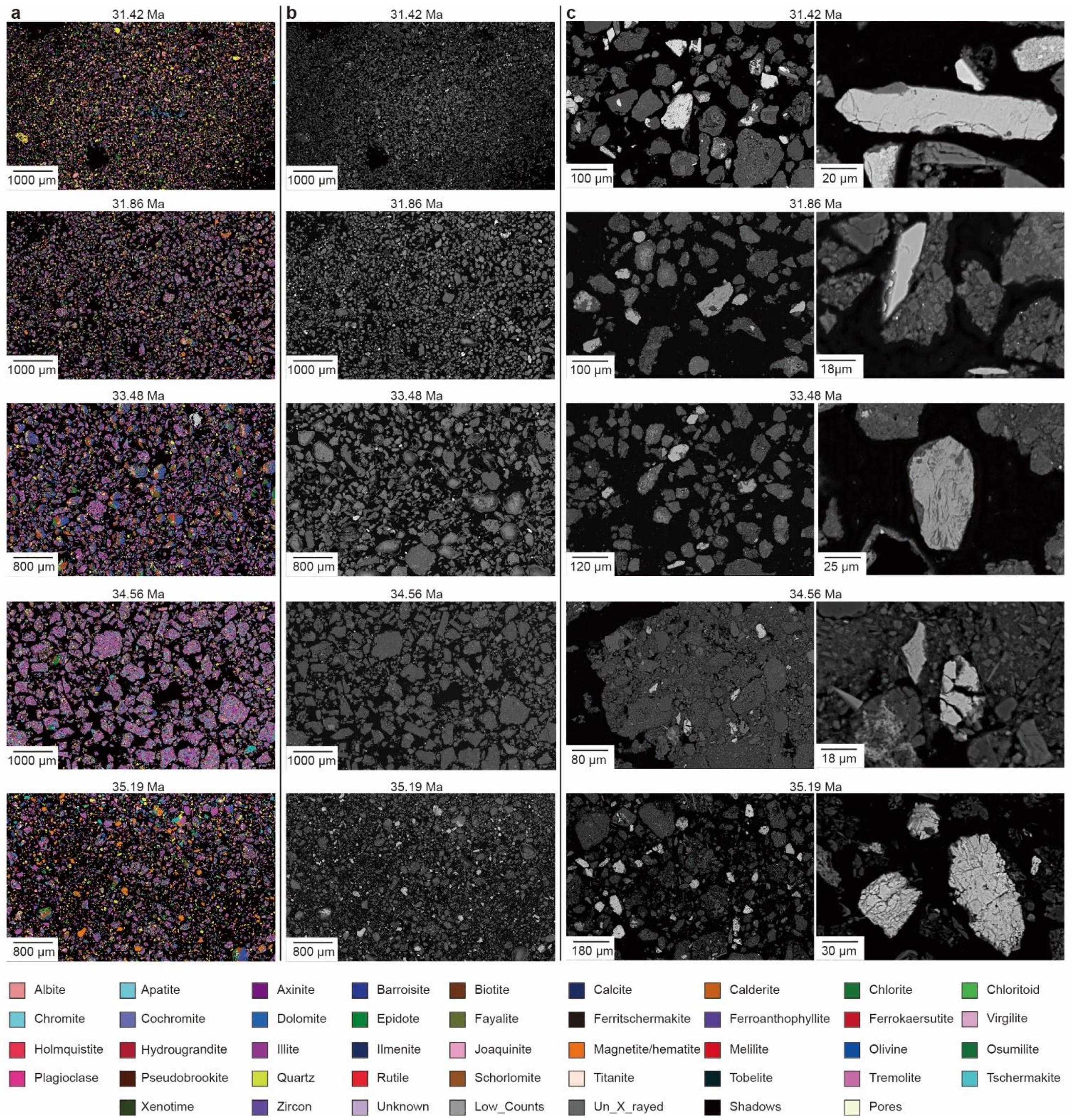

Supplementary Figure 11. Mineralogy and morphology of magnetic mineral extracts. (**a**, **b**) X-ray and original backscattered electron (BSE) images of magnetic extracts. Different minerals are recognized by the X-ray spectrum and are shown with different colours. (**c**) Enlarged BSE images of magnetite/hematite particles (bright) in the magnetic extracts.

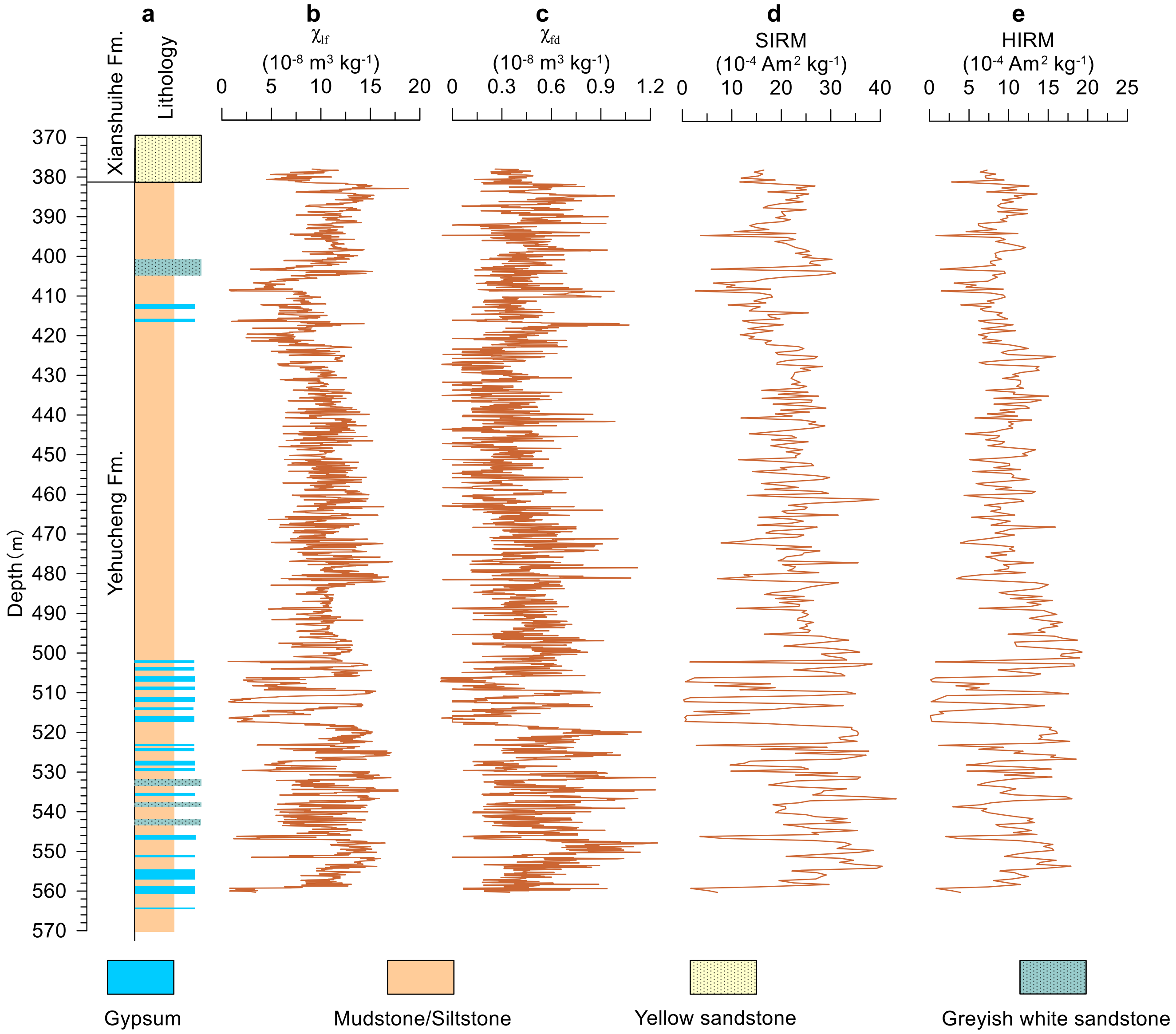

Supplementary Figure 12. Lithological and environmental magnetic data versus depth. (a–e) Changes of lithostratigraphy, low-frequency magnetic susceptibility (χ_{lf}), frequency-dependent magnetic susceptibility (χ_{fd}), saturation isothermal remanent magnetization (SIRM), and hard isothermal remanent magnetization (HIRM) in the Duitinggou section as a function of stratigraphic level. These parameters vary consistently through the studied section and reflect magnetic mineral concentration changes.

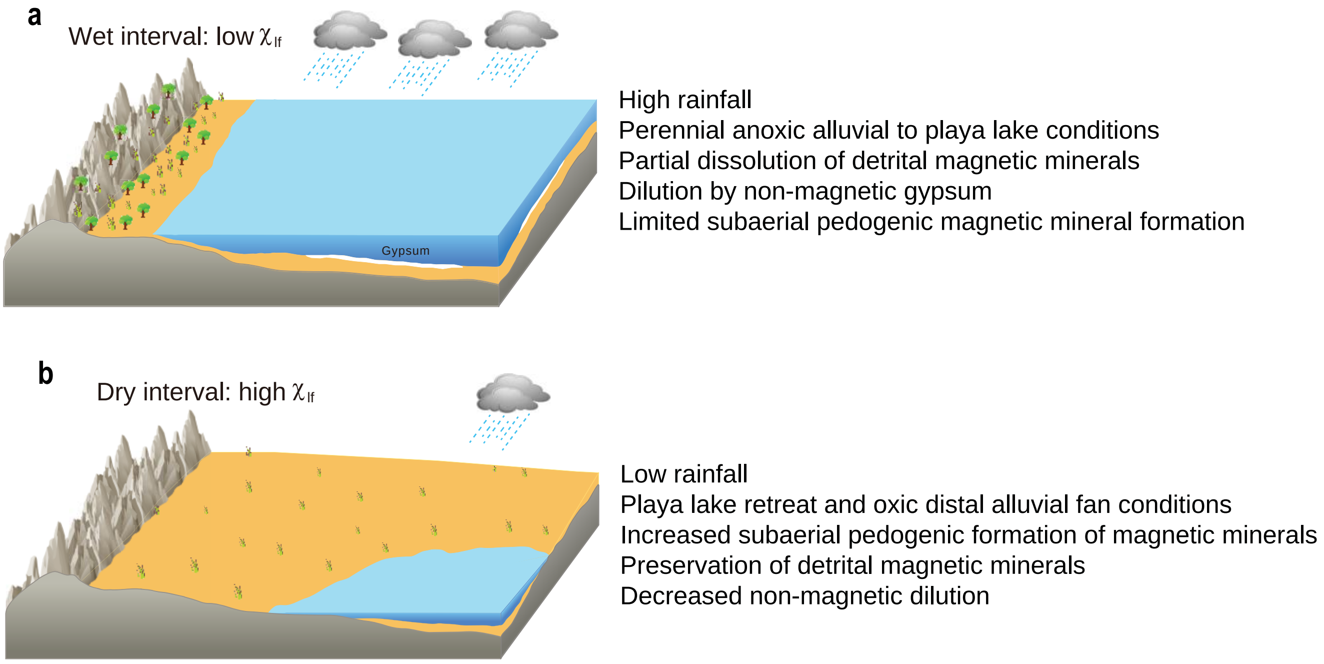

Supplementary Figure 13. Link between environmental interpretations and magnetic parameter variations. (**a**, **b**) Conceptual model of the processes that may have controlled detrital mineral transportation, subaerial pedogenic magnetic mineral formation during transportation and deposition, post-depositional magnetic mineral alteration, and non-magnetic dilution in the Lanzhou palaeolake during wet and low-χ_{lf} and dry and high-χ_{lf} phases. χ_{lf}: low-frequency magnetic susceptibility.

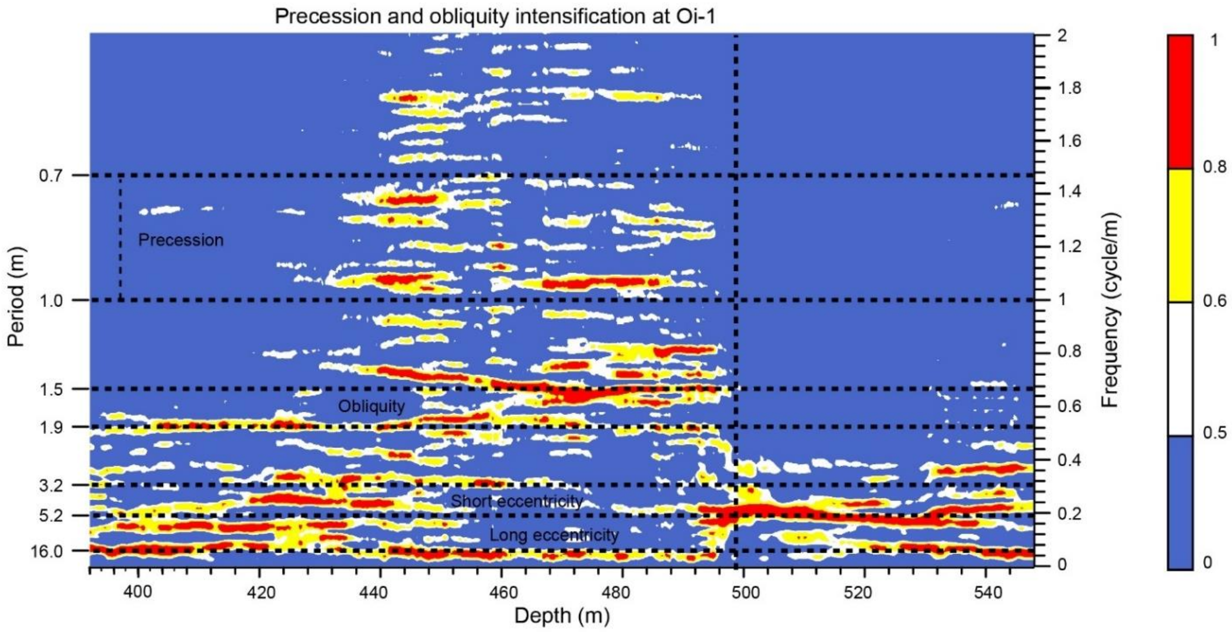

Supplementary Figure 14. Orbital features of the magnetic proxy record in the depth domain. Spectral evolution of the Duitinggou χ_{lf} record in the depth domain was calculated with a 28-m sliding window and 0.3-m sliding step. More saturated red colours represent higher spectral power. The distinct band with period of ~16 m corresponds to the long eccentricity (405 kyr) signal, the band with period between 3.2 and 5.2 m corresponds to the short eccentricity (95 kyr and 125 kyr) signal, the band with period between 1.5 and 1.9 m corresponds to the obliquity signal, and the band with period between 0.7 and 1 m corresponds to the precession (~19 kyr, ~23 kyr) signal. χ_{lf}: low-frequency magnetic susceptibility.

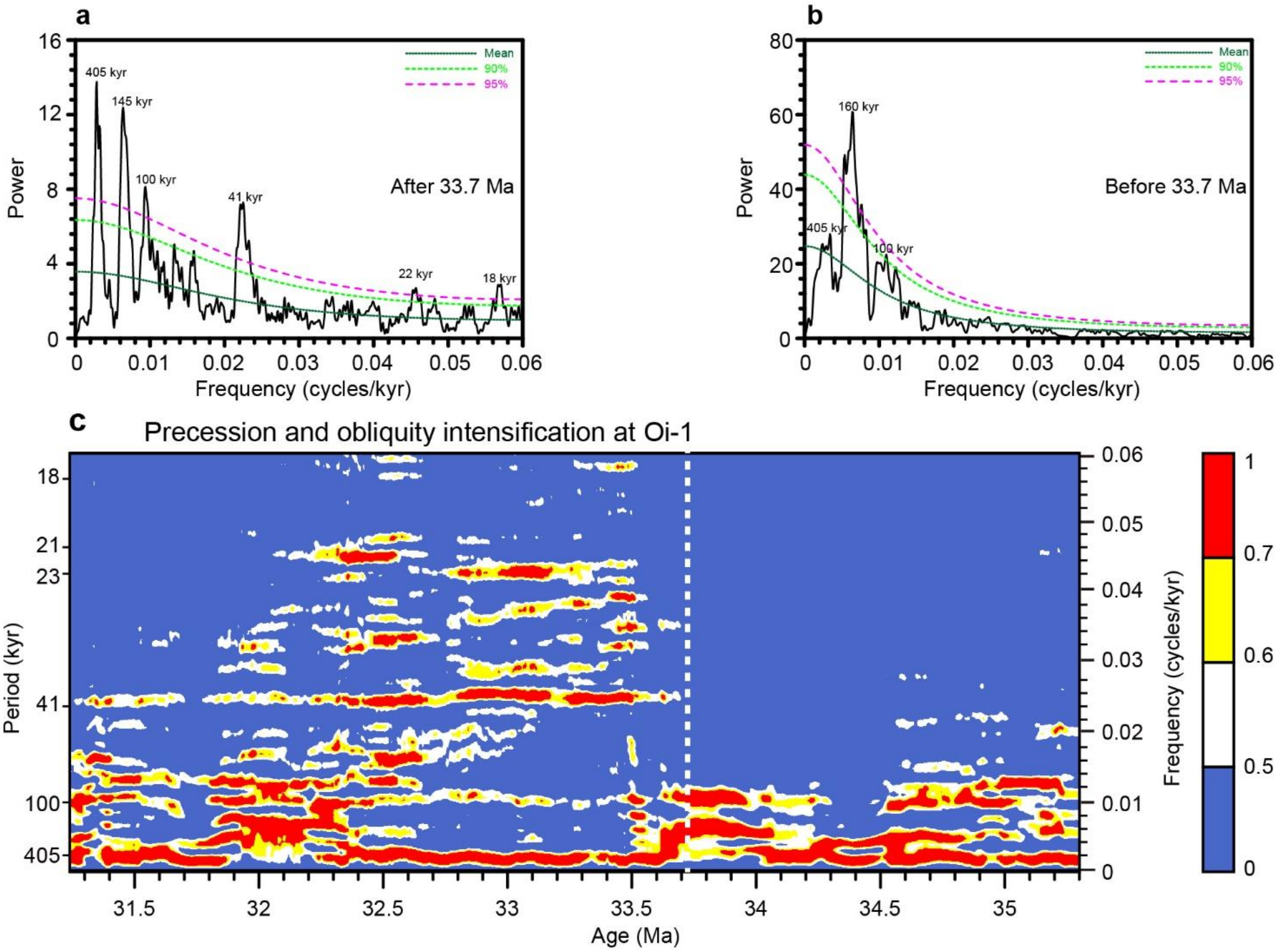

Supplementary Figure 15. Orbital features of the magnetic proxy record in the magnetochronology. (**a**, **b**) 2π-Multi-taper method (MTM) power spectrum and (**c**) spectral evolution of Duitinggou χ_{lf} in the untuned magnetochronology, with a 600-kyr sliding window and 8-kyr sliding step. Precession and obliquity cycles appear after ~33.7 Ma. χ_{lf}: low-frequency magnetic susceptibility.

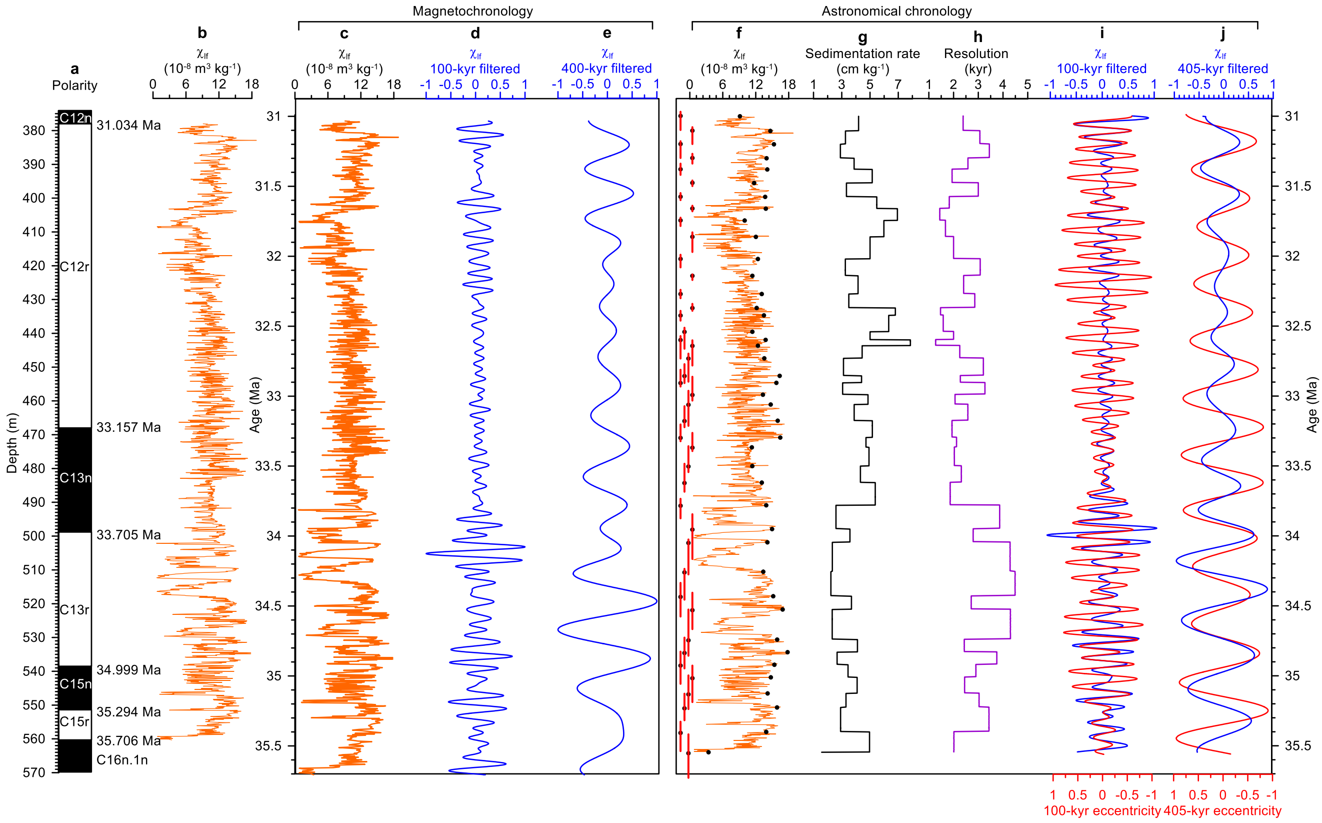

Supplementary Figure 16. Astronomical time scale construction. The astronomical chronology was generated by initially tuning 405-kyr components filtered from χ_{lf} and eccentricity[55], followed by fine-adjustment of individual 100-kyr cycles. (**a**) Polarity zones and (**b**) χ_{lf} record for the Duitinggou section as a function of stratigraphic level. Ages for magnetic reversal boundaries refer to the 2012 GPTS[35]. (**c**) χ_{lf} record plotted in the magnetochronology established by linear interpolation between ages of palaeomagnetic reversal boundaries[35]. Filtered (**d**) 100-kyr and (**e**) 405-kyr χ_{lf} components in the initial magnetochronological time scale. (**f**) Astronomically tuned χ_{lf} record. Black dots on the χ_{lf} record represent final age control points used to establish the astronomical time scale. Age control points with uncertainties (red lines) are also marked to the left of the χ_{lf} curve. (**g**) Linear sedimentation rates between age control points. (**h**) Resolution of the χ_{lf} record in the astronomical time scale. (**i**) 100-kyr and (**j**) 405-kyr components filtered from the tuned χ_{lf} record compared to those for orbital eccentricity[55]. χ_{lf}: low-frequency magnetic susceptibility; GPTS: geomagnetic polarity time scale.

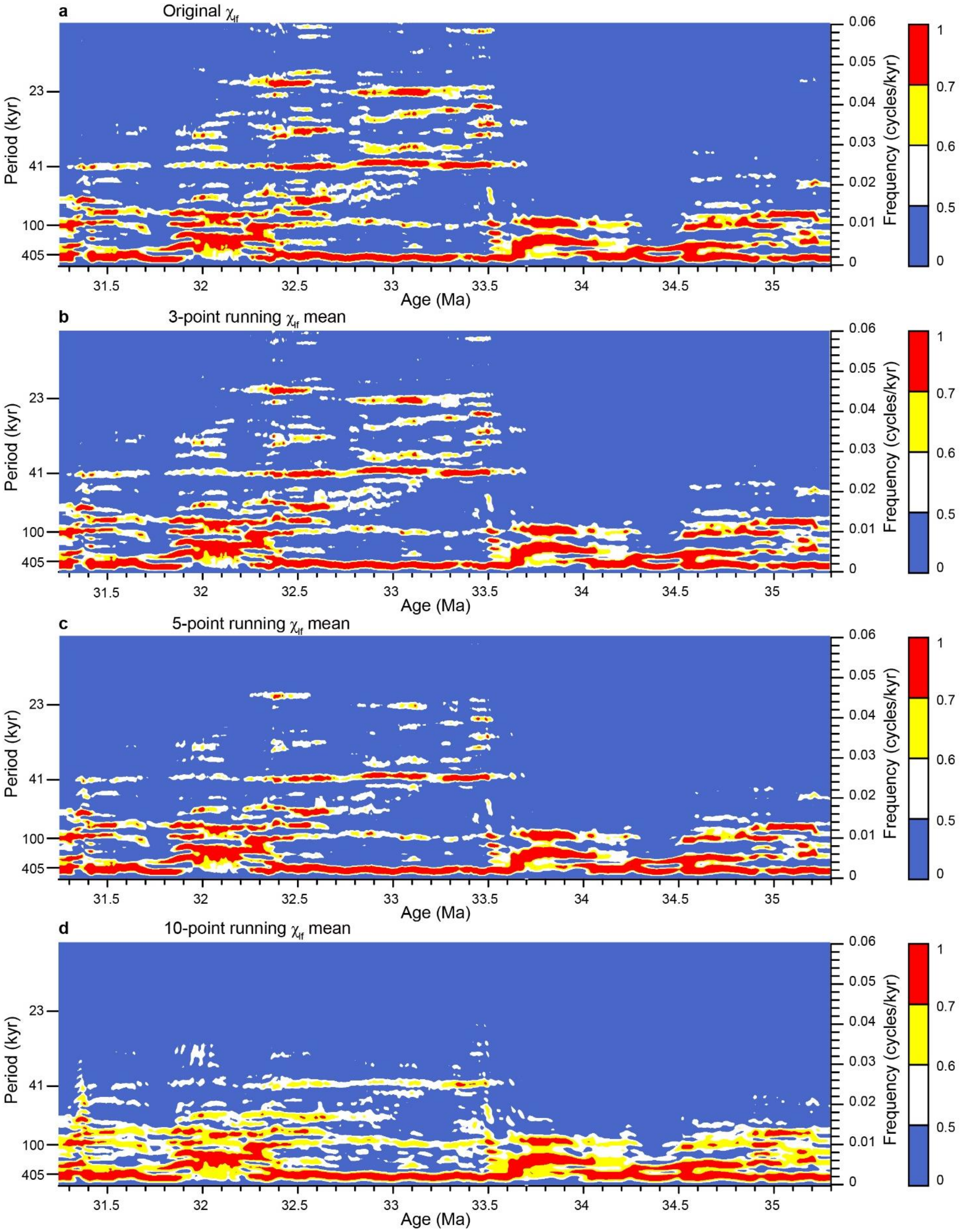

Supplementary Figure 17. Orbital features of the magnetic proxy record and their means. The original Duitinggou χ_{lf} evolutive spectrum is compared with that of its 3-point, 5-point, and 10-point running means in the eccentricity-based astronomical time scale. The evolutive spectrum was calculated with a 600-kyr sliding window and 2-kyr sliding step. χ_{lf}: low-frequency magnetic susceptibility.

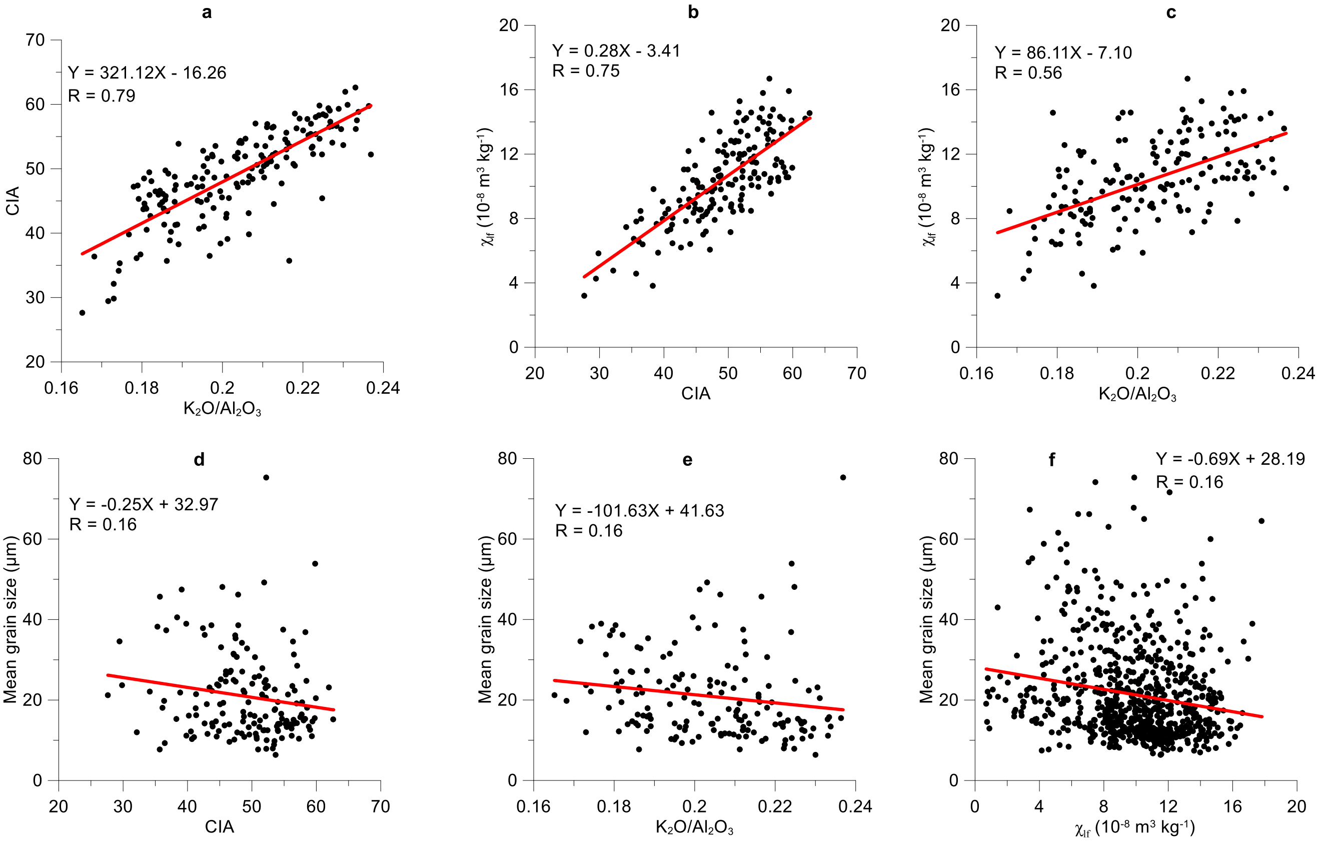

Supplementary Figure 18. Relationships among proxies for the Duitinggou section. Relationships of (**a**) CIA versus K_2O/Al_2O_3, (**b**) χ_{lf} versus CIA, (**c**) χ_{lf} versus K_2O/Al_2O_3, (**d**) mean grain size versus CIA, (**e**) mean grain size versus K_2O/Al_2O_3, and (**f**) mean grain size versus χ_{lf}. CIA: molar ratio of Al_2O_3 to $Al_2O_3 + CaO + Na_2O + K_2O$; χ_{lf}: low-frequency magnetic susceptibility.

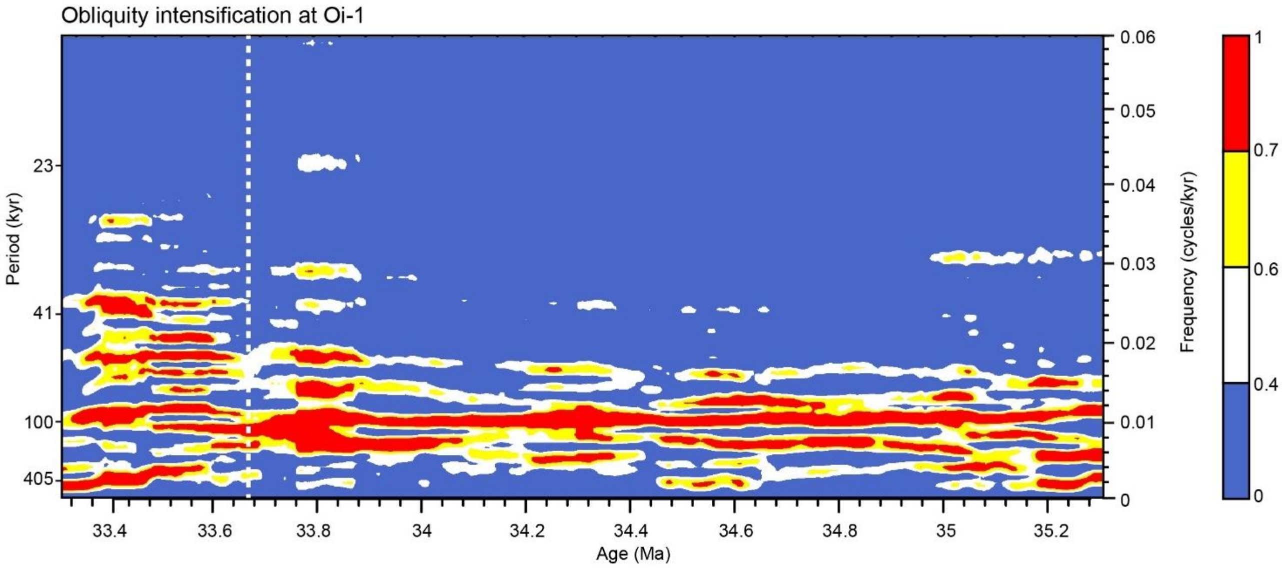

Supplementary Figure 19. Orbital features of the Xining Basin record. Spectral evolution of low-frequency magnetic susceptibility (χ_{lf}) from the Tashan section, Xining Basin[10] was calculated with a 600-kyr sliding window and 8-kyr sliding step.

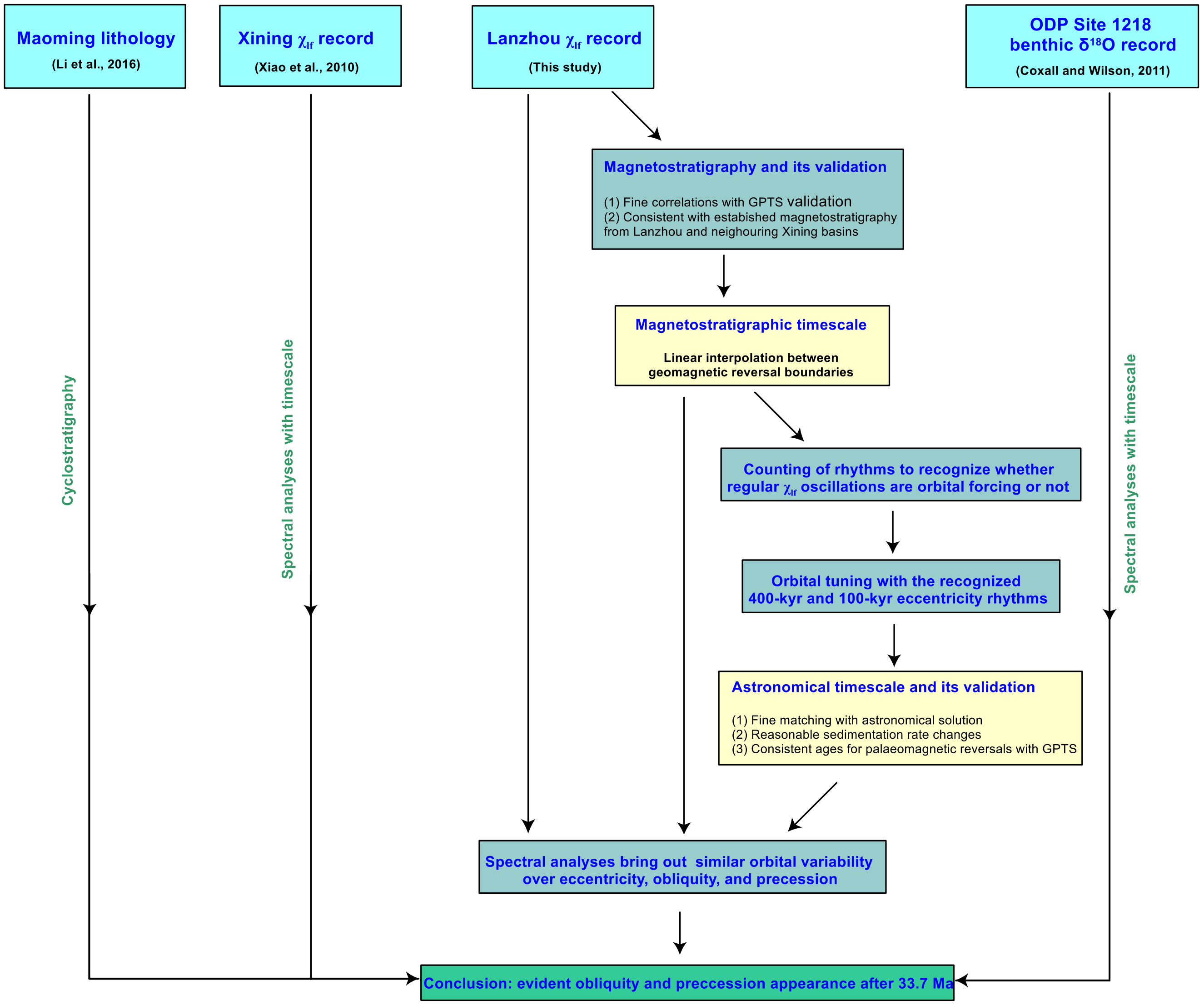

Supplementary Figure 20. Tuning strategy and other Eocene–Oligocene transition records. Flowchart of the tuning strategy used in this study and the observed orbital shift across the Eocene–Oligocene transition from Asian terrestrial and marine records testify to the robustness of the appearance of evident early Oligocene obliquity and precession cyclicity.

Supplementary Table 1. Age correlation points used to derive the adopted astronomical time scale.

Depth (m)	Depth uncertainty (m)	Age (Ma)	Age, - limit (Ma)	Age, + limit (Ma)	Negative uncertainty (- Myr)	Positive uncertainty (+ Myr)
378.0	0.05	31.000	30.970	31.100	0.030	0.100
382.4	0.05	31.105	31.080	31.200	0.025	0.095
385.5	0.05	31.200	31.180	31.300	0.020	0.100
388.4	0.05	31.300	31.270	31.340	0.030	0.040
391.5	0.05	31.380	31.340	31.420	0.040	0.040
396.5	0.05	31.477	31.460	31.500	0.017	0.023
399.8	0.05	31.576	31.550	31.600	0.026	0.024
404.4	0.05	31.660	31.640	31.680	0.020	0.020
410.3	0.05	31.745	31.725	31.785	0.020	0.040
417.3	0.05	31.862	31.830	31.970	0.032	0.108
425.2	0.05	32.020	31.990	32.080	0.030	0.060
429.1	0.05	32.140	32.130	32.170	0.010	0.030
434.5	0.05	32.270	32.240	32.300	0.030	0.030
438.0	0.05	32.370	32.340	32.390	0.030	0.020
441.6	0.05	32.423	32.390	32.460	0.033	0.037
449.0	0.05	32.540	32.510	32.660	0.030	0.120
451.9	0.05	32.598	32.570	32.730	0.028	0.132
455.2	0.05	32.640	32.600	32.780	0.040	0.140
459.2	0.05	32.730	32.695	32.895	0.035	0.165
463.1	0.05	32.855	32.770	32.905	0.085	0.050
465.3	0.05	32.905	32.810	32.930	0.095	0.025
467.9	0.05	32.990	32.860	33.030	0.130	0.040
471.3	0.05	33.060	32.960	33.210	0.100	0.150
475.8	0.05	33.176	33.070	33.220	0.106	0.044
482.0	0.05	33.296	33.200	33.320	0.096	0.024
485.3	0.05	33.366	33.260	33.390	0.106	0.024
491.9	0.05	33.500	33.355	33.540	0.145	0.040
497.0	0.05	33.618	33.480	33.680	0.138	0.062
505.7	0.05	33.780	33.730	33.870	0.050	0.090
510.1	0.05	33.950	33.840	34.160	0.110	0.210
513.5	0.05	34.045	34.020	34.270	0.025	0.225
518.4	0.05	34.255	34.230	34.450	0.025	0.195
522.3	0.05	34.430	34.330	34.570	0.100	0.140
525.8	0.05	34.525	34.400	34.660	0.125	0.135
530.8	0.05	34.740	34.520	34.850	0.220	0.110
534.5	0.05	34.830	34.720	34.940	0.110	0.110
536.9	0.05	34.920	34.820	35.050	0.100	0.130
540.0	0.05	35.010	34.920	35.180	0.090	0.170
544.7	0.05	35.125	34.990	35.230	0.135	0.105
548.0	0.05	35.225	35.120	35.310	0.105	0.085
553.1	0.05	35.400	35.320	35.530	0.080	0.130
560.3	0.05	35.545	35.410	35.720	0.135	0.175

Supplementary Table 2. Comparison of our astrochronologically estimated ages (Ma) for palaeomagnetic reversal boundaries with those estimated in previous studies. Ages of palaeomagnetic reversals in our astronomical time scale are consistent with their GPTS ages with uncertainties < 400 kyr. Tuning adjustments of the magnetochronology are accurate within a 405-kyr eccentricity cycle.

Magnetic reversal boundaries	CK95 age[56]	2004 GPTS[57]	HP2006 age[38]	2012 GPTS[35]	2020 GPTS[36]	This study
C12r–C12n	30.939	31.116	31.034	31.034	no data	31.000
C13n–C12r	33.058	33.266	33.157	33.157	33.076	32.992
C13r–C13n	33.545	33.738	33.705	33.705	33.675	33.652
C15n–C13r	34.655	34.782	35.126	34.999	34.875	34.966
C15r–C15n	34.940	35.043	35.254	35.294	35.199	35.345
C16n.1n–C15r	35.343	35.404	35.328	35.706	35.627	35.545

Supplementary References

1 McFadden, P. L. & McElhinny, M. W. Classification of the Reversal Test in Paleomagnetism. *Geophys. J. Int.* **103**, 725–729 (1990).

2 Zhang, P. et al. Magnetochronology of the Oligocene mammalian faunas in the Lanzhou Basin, Northwest China. *J. Asian Earth Sci.* **159**, 24–33 (2018).

3 Zhang, P., Ao, H., Dekkers, M. J., Li, Y. X. & An, Z. S. Late Oligocene–Early Miocene magnetochronology of the mammalian faunas in the Lanzhou Basin—environmental changes in the NE margin of the Tibetan Plateau. *Sci. Rep.* **6**, 38023, doi:38010.31038/srep38023 (2016).

4 Zhang, Y. B. et al. Cenozoic record of aeolian sediment accumulation and aridification from Lanzhou, China, driven by Tibetan Plateau uplift and global climate. *Glob. Planet. Change* **120**, 1–15 (2014).

5 Wang, W. T. et al. Pulsed growth of the West Qinling at ~30 Ma in northeastern Tibet: evidence from Lanzhou Basin magnetostratigraphy and provenance. *J. Geophys. Res.* **121**, 7754–7774 (2016).

6 Yue, L. P. et al. Magnetostratigraphy and paleoenvironmental record of Tertiary deposits of Lanzhou Basin. *Chinese Sci. Bull.* **46**, 770–773 (2001).

7 Dai, S. et al. Magnetostratigraphy of Cenozoic sediments from the Xining Basin: tectonic implications for the northeastern Tibetan Plateau. *J. Geophys. Res.* **111**, B11102, doi: 11110.11029/12005jb004187 (2006).

8 Dupont-Nivet, G. et al. Tibetan Plateau aridification linked to global cooling at the Eocene–Oligocene transition. *Nature* **445**, 635–638 (2007).

9 Zan, J. B., Fang, X. M., Yan, M. D., Zhang, W. L. & Lu, Y. Lithologic and rock magnetic evidence for the Mid-Miocene Climatic Optimum recorded in the sedimentary archive of the Xining Basin, NE Tibetan Plateau. *Palaeogeogr. Palaeoclimatol. Palaeoecol.* **431**, 6–14 (2015).

10 Xiao, G. Q., Abels, H. A., Yao, Z. Q., Dupont-Nivet, G. & Hilgen, F. J. Asian aridification linked to the first step of the Eocene–Oligocene climate Transition (EOT) in obliquity-dominated terrestrial records (Xining Basin, China). *Clim. Past* **6**, 501–513 (2010).

11 Xiao, G. Q. et al. Evidence for northeastern Tibetan Plateau uplift between 25 and 20 Ma in

the sedimentary archive of the Xining Basin, Northwestern China. *Earth Planet. Sci. Lett.* **317**, 185–195 (2012).

12 Fang, X. M. et al. Cenozoic magnetostratigraphy of the Xining Basin, NE Tibetan Plateau, and its constraints on paleontological, sedimentological and tectonomorphological evolution. *Earth-Sci. Rev.* **190**, 460–485 (2019).

13 Chang, L. et al. Coupled microbial bloom and oxygenation decline recorded by magnetofossils during the Palaeocene–Eocene Thermal Maximum. *Nat. Commun.* **9**, 4007, doi: 4010.1038/s41467-41018-06472-y (2018).

14 Chang, L., Heslop, D., Roberts, A. P., Rey, D. & Mohamed, K. J. Discrimination of biogenic and detrital magnetite through a double Verwey transition temperature. *J. Geophys. Res.* **121**, 3–14 (2016).

15 Özdemir, Ö. & Dunlop, D. J. Hallmarks of maghemitization in low-temperature remanence cycling of partially oxidized magnetite nanoparticles. *J. Geophys. Res.* **115**, B02101, doi:02110.01029/02009JB006756 (2010).

16 Egli, R., Chen, A. P., Winklhofer, M., Kodama, K. P. & Horng, C. S. Detection of noninteracting single domain particles using first-order reversal curve diagrams. *Geochem. Geophys. Geosyst.* **11**, Q01Z11, doi:10.1029/2009GC002916 (2010).

17 Ao, H., Deng, C. L., Dekkers, M. J. & Liu, Q. S. Magnetic mineral dissolution in Pleistocene fluvio-lacustrine sediments, Nihewan Basin (North China). *Earth Planet. Sci. Lett.* **292**, 191–200 (2010).

18 Roberts, A. P., Pike, C. R. & Verosub, K. L. First-order reversal curve diagrams: a new tool for characterizing the magnetic properties of natural samples. *J. Geophys. Res.* **105**, 28461–28475 (2000).

19 Roberts, A. P., Heslop, D., Zhao, X. & Pike, C. R. Understanding fine magnetic particle systems through use of first-order reversal curve diagrams. *Rev. Geophys.* **52**, 557–602 (2014).

20 Pike, C. R., Roberts, A. P. & Verosub, K. L. First-order reversal curve diagrams and thermal relaxation effects in magnetic particles. *Geophys. J. Int.* **145**, 721–730 (2001).

21 Zhang, C. X. & Guo, Z. T. Clay mineral changes across the Eocene–Oligocene transition in the sedimentary sequence at Xining occurred prior to global cooling. *Palaeogeogr. Palaeoclimatol. Palaeoecol.* **411**, 18–29 (2014).

22 Fang, X. M. et al. An Eocene–Miocene continuous rock magnetic record from the sediments in the Xining Basin, NW China: indication for Cenozoic persistent drying driven by global cooling and Tibetan Plateau uplift. *Geophys. J. Int.* **201**, 78–89 (2015).

23 Page, M. et al. Synchronous cooling and decline in monsoonal rainfall in northeastern Tibet during the fall into the Oligocene icehouse. *Geology* **47**, 203–206 (2019).

24 Ortega, B., Caballero, M., Lozano, S., Vilaclara, G. & Rodriguez, A. Rock magnetic and geochemical proxies for iron mineral diagenesis in a tropical lake: Lago Verde, Los Tuxtlas, East-Central Mexico. *Earth Planet. Sci. Lett.* **250**, 444–458 (2006).

25 Snowball, I. F. Geochemical control of magnetite dissolution in subarctic lake sediments and the implications for environmental magnetism. *J. Quat. Sci.* **8**, 339–346 (1993).

26 Geiss, C. E., Umbanhowar, C. E., Camill, P. & Banerjee, S. K. Sediment magnetic properties reveal Holocene climate change along the Minnesota prairie-forest ecotone. *J. Paleolimnol.* **30**, 151–166 (2003).

27 Zhang, J. et al. Magnetostratigraphic age and monsoonal evolution recorded by the thickest Quaternary loess deposit of the Lanzhou region, western Chinese Loess Plateau. *Quat. Sci. Rev.* **139**, 17–29 (2016).

28 Peng, X. Z., Ao, H., Xiao, G. Q., Qiang, X. K. & Sun, Q. The Early–Middle Pleistocene transition of Asian summer monsoon. *Palaeogeogr. Palaeoclimatol. Palaeoecol.* **545**, 109636 (2020).

29 Licht, A. et al. Asian monsoons in a late Eocene greenhouse world. *Nature* **513**, 501–506 (2014).

30 Li, J. X. et al. Global cooling and enhanced Eocene Asian mid-latitude interior aridity. *Nat. Commun.* **9**, 3026, doi: 3010.1038/s41467-41018-05415-x (2018).

31 Wang, C. S. et al. Outward-growth of the Tibetan Plateau during the Cenozoic: a review. *Tectonophysics* **621**, 1–43 (2014).

32 Lisiecki, L. E. & Raymo, M. E. A Pliocene–Pleistocene stack of 57 globally distributed benthic $\delta^{18}O$ records. *Paleoceanography* **20**, PA1003, doi:1010.1029/2004PA001071 (2005).

33 Sun, Y. B., Clemens, S. C., An, Z. S. & Yu, Z. W. Astronomical timescale and palaeoclimatic implication of stacked 3.6-Myr monsoon records from the Chinese Loess Plateau. *Quat. Sci. Rev*. **25**, 33–48 (2006).

34 Herb, C. et al. Orbitally tuned age model for the late Pliocene–Pleistocene lacustrine succession of drill core SG-1 from the western Qaidam Basin (NE Tibetan Plateau). *Geophys. J. Int.* **200**, 35–51 (2015).

35 Vandenberghe, N., Hilgen, F. J. & Speijer, R. P. The Paleogene Period. In *The Geologic Time Scale* (eds Gradstein, F. M., Ogg, J. G., Schmitz, M. & Ogg, G.). 855–922 (Elsevier, Amsterdam, 2012).

36 Malinverno, A., Quigley, K. W., Staro, A. & Dyment, J. A late Cretaceous–Eocene geomagnetic polarity timescale (MQSD20) that steadies spreading rates on multiple mid-ocean ridge flanks. *J. Geophys. Res.* **125**, e2020JB020034, doi.org/020010.021029/022020JB020034 (2020).

37 Toby, S. C., Duller, R. A., De Angelis, S. & Straub, K. M. A stratigraphic framework for the preservation and shredding of environmental signals. *Geophys. Res. Lett.* **46**, 5837–5845 (2019).

38 Pälike, H. et al. The heartbeat of the Oligocene climate system. *Science* **314**, 1894–1898 (2006).

39 Levy, R. H. et al. Antarctic ice-sheet sensitivity to obliquity forcing enhanced through ocean connections. *Nat. Geosci.* **12**, 132–137 (2019).

40 Coxall, H. K., Wilson, P. A., Pälike, H., Lear, C. H. & Backman, J. Rapid stepwise onset of Antarctic glaciation and deeper calcite compensation in the Pacific Ocean. *Nature* **433**, 53–57 (2005).

41 De Vleeschouwer, D., Vahlenkamp, M., Crucifix, M. & Pälike, H. Alternating Southern and Northern Hemisphere climate response to astronomical forcing during the past 35 m.y. *Geology* **45**, 375–378 (2017).

42 Pälike, H., Frazier, J. & Zachos, J. C. Extended orbitally forced palaeoclimatic records from the equatorial Atlantic Ceara Rise. *Quat. Sci. Rev*. **25**, 3138–3149 (2006).

43 Erhardt, A. M., Pälike, H. & Paytan, A. High-resolution record of export production in the eastern equatorial Pacific across the Eocene–Oligocene transition and relationships to global climatic records. *Paleoceanography* **28**, 130–142 (2013).

44 Pälike, H., Shackleton, N. J. & Rohl, U. Astronomical forcing in Late Eocene marine sediments. *Earth Planet. Sci. Lett.* **193**, 589–602 (2001).

45 Westerhold, T. et al. Orbitally tuned timescale and astronomical forcing in the middle Eocene to early Oligocene. *Clim. Past* **10**, 955–973 (2014).

46 Su, Q. D. et al. Detection of strong precession cycles from the late Pliocene sedimentary records of northeastern Tibetan Plateau. *Geochem. Geophys. Geosyst.* **20**, doi: 10.1029/2019GC008447 (2019).

47 White, D. S. & Miller, M. F. Benthic invertebrate activity in lakes: linking present and historical bioturbation patterns. *Aquat. Biol.* **2**, 269–277 (2008).

48 Orme, L. C. et al. Investigating the maximum resolution of mu XRF core scanners: a 1800 year storminess reconstruction from the Outer Hebrides, Scotland, UK. *Holocene* **26**, 235–247 (2016).

49 Boudreau, B. P. Mean mixed depth of sediments: the wherefore and the why. *Limnol. Oceanogr.* **43**, 524–526 (1998).

50 Teal, L. R., Bulling, M. T., Parker, E. R. & Solan, M. Global patterns of bioturbation intensity and the mixed depth of marine soft sediments. *Aquat. Biol.* **2**, 207–218 (2008).

51 Li, Y. X. et al. Terrestrial responses of low-latitude Asia to the Eocene–Oligocene climate transition revealed by integrated chronostratigraphy. *Clim. Past* **12**, 255–272 (2016).

52 Coxall, H. K. & Wilson, P. A. Early Oligocene glaciation and productivity in the eastern equatorial Pacific: Insights into global carbon cycling. *Paleoceanography* **26**, PA2221, doi:2210.1029/2010PA002021 (2011).

53 Zijderveld, J. D. A. AC demagnetization of rocks: analysis of results. In *Methods in Paleomagnetism* (eds Collinson, D.W., Creer, K.M. & Runcorn S.K.) 254–286 (Elsevier, Amsterdam, 1967).

54 Kruiver, P. P., Dekkers, M. J. & Heslop, D. Quantification of magnetic coercivity components by the analysis of acquisition curves of isothermal remanent magnetisation. *Earth Planet. Sci. Lett.* **189**, 269–276 (2001).

55 Laskar, J., Fienga, A., Gastineau, M. & Manche, H. La2010: a new orbital solution for the long-term motion of the Earth. *Astron. Astrophys.* **532**, A89, doi:10.1051/0004-6361/201116836 (2011).

56 Cande, S. C. & Kent, D. V. Revised calibration of the geomagnetic polarity timescale for the late Cretaceous and Cenozoic. *J. Geophys. Res.* **100**, 6093–6095 (1995).

57 Luterbacher, H. P. et al. The Paleogene Period. In *A Geologic Time Scale 2004* (eds Gradstein, F. M. Ogg J. G., & Smith A. G.) 384–408 (Cambridge University Press, Cambridge, 2004).
